# *Arabidopsis* vegetative actin isoforms, AtACT2 and AtACT7, generate distinct filament arrays in living plant cells

**DOI:** 10.1038/s41598-018-22707-w

**Published:** 2018-03-12

**Authors:** Saku T. Kijima, Christopher J. Staiger, Kaoru Katoh, Akira Nagasaki, Kohji Ito, Taro Q. P. Uyeda

**Affiliations:** 1 Biomedical Research Institute, National Institute of Advanced Industrial Science and Technology, Ibaraki, 305–8565 Japan; 2 Graduate School of Life and Environmental Sciences, University of Tsukuba, Ibaraki, 305–8572 Japan; 3 Department of Botany and Plant Pathology, Purdue University, IN, 47907 USA; 4 Department of Biology, Graduate School of Science, Chiba University, Chiba, 263–8522 Japan; 5 Department of Physics, Faculty of Science and Engineering, Waseda University, Tokyo, 169–8555 Japan

## Abstract

Flowering plants express multiple actin isoforms. Previous studies suggest that individual actin isoforms have specific functions; however, the subcellular localization of actin isoforms in plant cells remains obscure. Here, we transiently expressed and observed major *Arabidopsis* vegetative actin isoforms, AtACT2 and AtACT7, as fluorescent-fusion proteins. By optimizing the linker sequence between fluorescent protein and actin, we succeeded in observing filaments that contained these expressed actin isoforms fused with green fluorescent protein (GFP) in *Arabidopsis* protoplasts. Different colored fluorescent proteins fused with AtACT2 and AtACT7 and co-expressed in *Nicotiana benthamiana* mesophyll cells co-polymerized in a segregated manner along filaments. In epidermal cells, surprisingly, AtACT2 and AtACT7 tended to polymerize into different types of filaments. AtACT2 was incorporated into thinner filaments, whereas AtACT7 was incorporated into thick bundles. We conclude that different actin isoforms are capable of constructing unique filament arrays, depending on the cell type or tissue. Interestingly, staining patterns induced by two indirect actin filament probes, Lifeact and mTalin1, were different between filaments containing AtACT2 and those containing AtACT7. We suggest that filaments containing different actin isoforms bind specific actin-binding proteins *in vivo*, since the two probes comprise actin-binding domains from different actin-binding proteins.

## Introduction

In plant cells, actin participates in numerous activities such as cell division and morphogenesis, tip growth, movement and repositioning of organelles, cytoplasmic streaming, fertilization, hormone transport and responses to external signals^1–5^. Actin achieves these diverse and complex functions by interacting with various actin-binding proteins (ABPs). Angiosperms have multiple actin and ABP isoforms. For example, the model plant, *Arabidopsis thaliana*, has eight actin isoforms that are grouped into two classes, vegetative (AtACT2, 7 and 8) and reproductive (AtACT1, 3, 4, 11 and 12), according to their expression patterns^6,7^. Previous genetic studies showed distinct expression patterns of actin isoforms and their possible specific functions^8^. Loss of function of the reproductive AtACT11 caused delayed pollen germination and enhanced pollen tube growth, accompanied by an increase in the rate of actin turnover^9^. A defect in root hair growth caused by the AtACT2 knock-out was not complemented by over-expression of AtACT7^10^. Ectopic expression of a reproductive actin isoform in vegetative tissues caused abnormal growth by accumulating aberrant bundled filaments^11^. These phenotypes, resulting from the ectopic expression of a reproductive actin, were suppressed by the ectopic co-expression of corresponding reproductive ABPs, profilin or actin depolymerizing factor (ADF/cofilin)^12,13^. These reports imply that individual actin isoforms interact with specific ABPs to fulfill specific cellular functions. Moreover, different *Arabidopsis* actin isoforms have been shown to have significantly different biochemical properties, such as assembly kinetics or binding to phalloidin and profilin^14^. These results suggest that plants have developed a mechanism to diversify actin cytoskeletal function by expressing multiple, functionally non-equivalent actin isoforms. However, it is unclear how multiple actin isoforms perform specific functions in plant cells. One approach to address this issue is to evaluate the subcellular distribution of individual actin isoforms within a cell.

At present, little is known about the localization of individual actin isoforms in plants, for several technical reasons. First, a high sequence similarity among actin isoforms makes it difficult to distinguish each actin isoform immunochemically^8^. Isoform-specific anti-*Arabidopsis* actin monoclonal antibodies were developed by Kandasamy *et al*.^15,16^, but they are not capable to distinguish each actin isoform by double indirect immunofluorescent staining because both antibodies are mouse IgG1. Second, in order to observe the actin cytoskeleton in living plant cells, Ren *et al*. and Jing *et al*. microinject fluorescently labeled actin into stamen hair cells^17,18^. They demonstrated that injected vertebrate skeletal muscle actin showed aberrant massive polymerization. In contrast, injected pollen actin showed normal actin organization. However, microinjection into plant cells is technically more difficult than into animal cells because of the thick cell walls and the large turgor pressure of plant cells^19^. In addition, microinjection limits the observation period, as well as the cell types and number of cells that can be observed. Third, expression of green fluorescent protein (GFP)-fusion protein is a standard method to detect the intracellular localization of specific protein isoforms. Many studies successfully visualized actin filaments in other live eukaryotic cells, including *Dictyostelium discoideum*^20^ and mammalian cells^21,22^, by expression of GFP-actin. In plant cells, two studies reported expression of actin-GFP in *Nicotiana tabacum* BY2 cells and protoplasts, respectively, but no long filamentous structures were observed even though localized GFP fluorescence was detected^23,24^. For these reasons, GFP fused with the actin-binding domain (ABD) of mouse talin1 (GFP-mTalin1), was the first GFP-based probe to observe actin filaments in living plant cells^25^. Thereafter, GFP probes fused with ABDs of various ABPs, such as Fimbrin1 from *Arabidopsis*^26^ and Lifeact derived from the yeast Abp140p^27^, in addition to mTalin1 mentioned above, have been widely used to visualize the organization of actin filaments in living plant cells^28,29^. When this method is used, however, it is difficult to distinguish between different actin isoforms. For these reasons, it is currently poorly understood how multiple actin isoforms behave in living plant cells.

The purpose of this study is to reveal the subcellular distribution of different plant actin isoforms and to clarify whether different actin isoforms are incorporated into the same filaments or not. Here, we focus on two major *Arabidopsis* vegetative actin isoforms, AtACT2 and AtACT7. Interestingly, there are 28 amino acid substitutions between AtACT2 and AtACT7, which are scattered throughout the molecule^6^. This is in sharp contrast to the difference between human cytoplasmic β and γ actin isoforms, the two major actin isoforms in non-muscle cells, that have only four conservative changes at the N-terminus^30^. Furthermore, these two *Arabidopsis* vegetative actin isoforms have distinct biochemical properties^14^. As a first step, we attempted to construct plasmids for transient expression of GFP fused with a vegetative actin isoform that is able to form filaments in living plant cells, by optimizing the linker sequence between actin and fluorescent protein as well as the location of GFP relative to actin. Optimized GFP-actin isoforms were incorporated into filamentous structures in *Arabidopsis* protoplasts. We then compared the distribution of the two major vegetative actin isoforms, AtACT2 and AtACT7, in *N*. *benthamiana* leaf cells. The results revealed that different actin isoforms form unique filament arrays in leaf epidermal and mesophyll sponge cells, providing platforms to understand different functions of actin isoforms in plant cells.

## Results

### Design of fluorescent-fusion proteins with *Arabidopsis* actin isoforms and expression in *Arabidopsis* protoplasts

**Figure 1:**
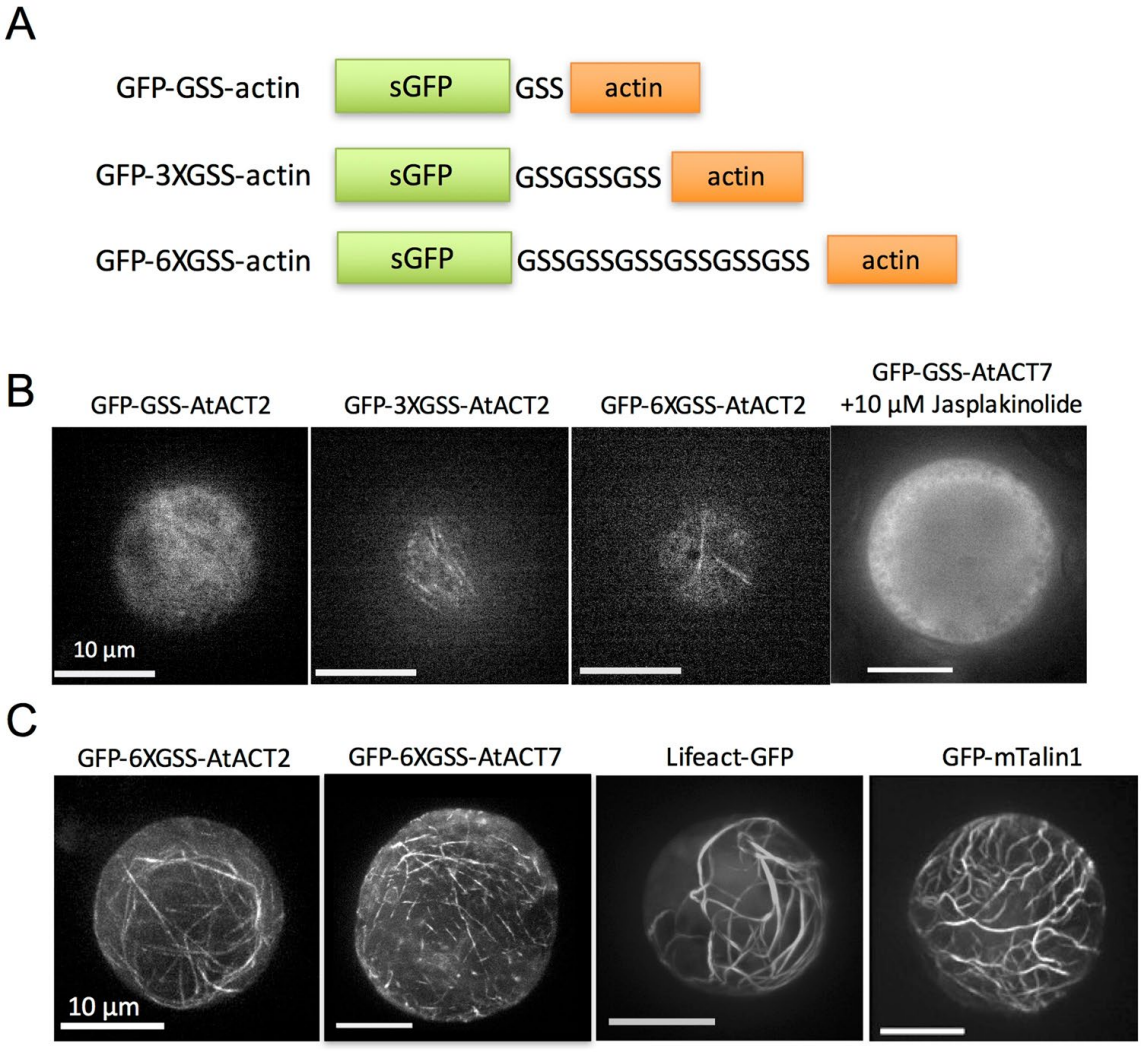
Design and expression of GFP-actin in *Arabidopsis* protoplasts. (**A**) Optimization of the linker length between GFP and actin. Three different linkers, Gly-Ser-Ser (GSS) unit repeated from one to six times, were inserted between N-terminal GFP and C-terminal actin. (**B**) Optical section images of *Arabidopsis* protoplasts transiently expressing each GFP-GSS-AtACT2, GFP-3XGSS-AtACT2, GFP-6XGSS-AtACT2 or GFP-GSS-AtACT7. The addition of 10 µM Jasplakinolide did not induce filamentous structures of GFP-GSS-AtACT7. (**C**) Z-series projections of protoplasts transiently expressing GFP-6XGSS-AtACT2 or GFP-6XGSS-AtACT7, or indirect actin filament reporters Lifeact-GFP or GFP-mTalin.

Actin directly fused to a fluorescent protein is useful to distinguish the localization of individual actin isoforms in eukaryotic cells, although visualization of long actin filaments by expressing GFP-actin has not been reported in plant cells. When designing GFP-actin constructs that form filaments *in vivo*, we recognized two variables: (i) the length of the in-frame linker between GFP and actin; and (ii) the position of GFP, either fused to the C-terminus or the N-terminus of actin. Regarding the position of GFP relative to actin, we recently reported that filaments of GFP fused to the C-terminus of actin using the long 6XGSS linker were difficult to observe, due to high cytoplasmic fluorescence from G-actin^31^. Thus, we optimized the linker between the N-terminal GFP moiety and the C-terminal actin moiety, using one to six repeats of the Gly-Ser-Ser (GSS) unit (Fig. 1A). GFP-AtACT2 with a short linker (GFP-GSS-AtACT2) expressed transiently in the protoplasts from *Arabidopsis* T87 cultured cells displayed only a diffuse distribution throughout the cytoplasm (Fig. 1B). Even in the presence of the actin-polymerizing drug, Jasplakinolide, a filamentous structure of GFP-actin with the short linker was not visualized. In contrast, GFP-actin with the intermediate or long linker (GFP-3XGSS-AtACT2 or GFP-6XGSS-AtACT2) showed filamentous structures (Fig. 1B). This result demonstrated that GFP-actin requires a linker composed of at least nine amino acid residues in order to polymerize in plant cells. In the Z-stack projections, both GFP-6XGSS-AtACT2 and GFP-6XGSS-AtACT7 were incorporated into long filamentous structures (Fig. 1C). The generic actin filament probes, Lifeact-GFP and GFP-mTalin1, also decorated filamentous structures (Fig. 1C). Based on these results, we decided to employ a fusion protein composed of fluorescent protein, GFP or TagRFP (red fluorescent protein), directly fused to the N-terminus of actin via the long linker, which we simply refer to hereafter as GFP- or TagRFP-actin. Note that even though expression of GFP-actin could be visualized in filaments, high cytoplasmic fluorescence was observed in some cells. This may be due to over-expression, because the total fluorescence intensity of these cells was higher than that in cells displaying recognizable filaments.

**Figure 2:**
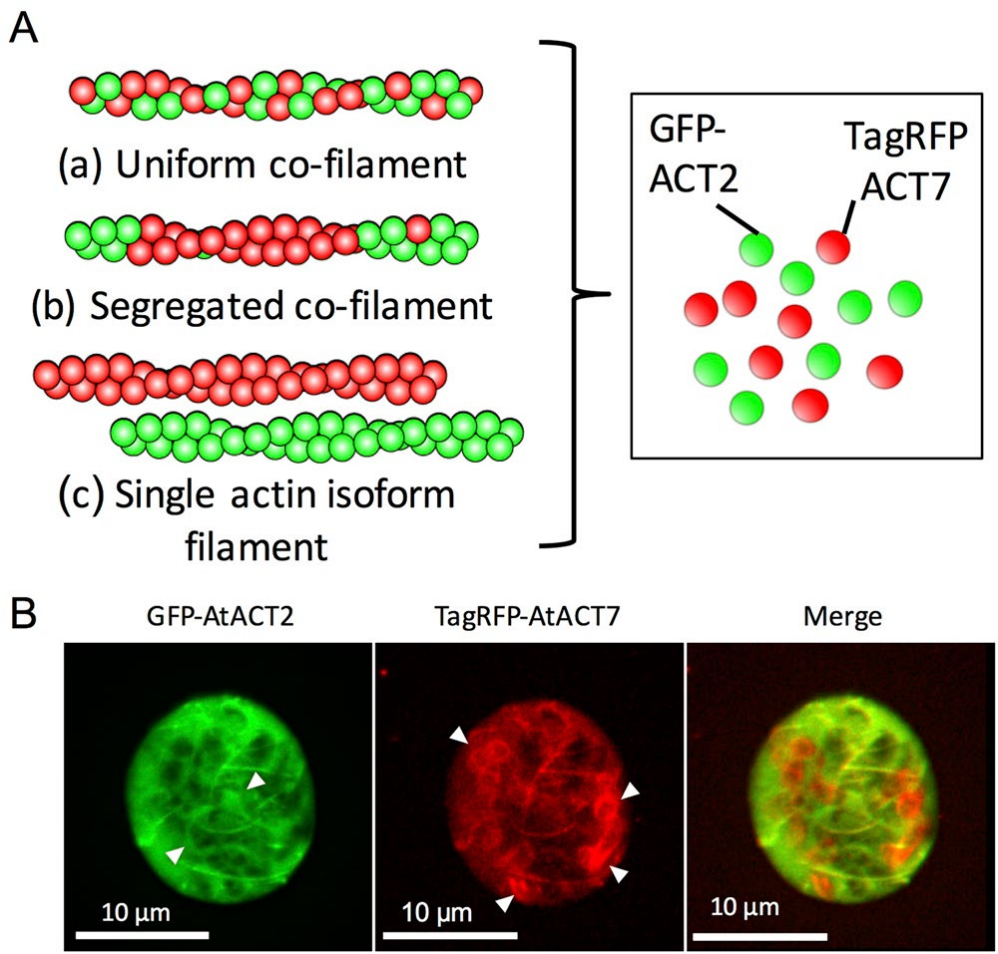
Distribution of GFP-AtACT2 and TagRFP-AtACT7 in *Arabidopsis* protoplasts. (**A**) Possible polymerization patterns of different actin isoforms. In order to distinguish different actin isoforms, two actin isoforms were translationally fused to a different fluorescent protein, GFP or TagRFP. (**B**) Z-series projection of a protoplast transiently co-expressing GFP-AtACT2 (left) and TagRFP-AtACT7 (middle), both with the long linker. The merged image is shown on the right. White arrowheads indicate filaments observed only in either the red or green channel.

Regarding the relationship between filaments made of the two GFP-actin isoforms, the following three polymerization patterns are envisaged (Fig. 2A). In the first case, different actin isoforms uniformly co-polymerize along the filaments; in the second case, different isoforms co-polymerize in a segregated manner; and in the third case, different actin isoforms do not co-polymerize but form separate filaments consisting of a single actin isoform. To distinguish among these possibilities, GFP-AtACT2 and TagRFP-AtACT7 were co-expressed in protoplasts. Interestingly, while some filaments were observed in both green and red channels, other filaments were observed in only one channel, suggesting that those filaments contain either GFP-AtACT2 or TagRFP-AtACT7, but not both (Fig. 2B, white arrowheads). Thus, protoplasts contained three types of actin filaments: those that contained both GFP-AtACT2 and TagRFP-AtACT7, those that contained only GFP-AtACT2, and those that contained only TagRFP-AtACT7. However, the fraction of cells co-expressing both GFP-actin and TagRFP-actin was very small in protoplasts.

### Distribution of AtACT2 and AtACT7 in leaf mesophyll cells

To perform a detailed assessment of the relationship between the two actin isoforms, we switched to a different, transient expression system, the *Agrobacterium* infiltration method using *N*. *benthamiana* leaves. Gunning *et al*. showed that plant actin isoforms are often more closely related to those of other species than within the same species^32^. Indeed, in a phylogenic tree of the amino acid sequences of *A*. *thaliana* and *N*. *benthamiana* actin isoforms, the actin isoforms are divided into three groups, and both *A*. *thaliana* and *N*. *benthamiana* have isoforms in each group (Supplemental Fig. 1). For these reasons, it seemed legitimate to express *Arabidopsis* actin isoforms in *N*. *benthamiana* cells for localization studies.

**Figure 3:**
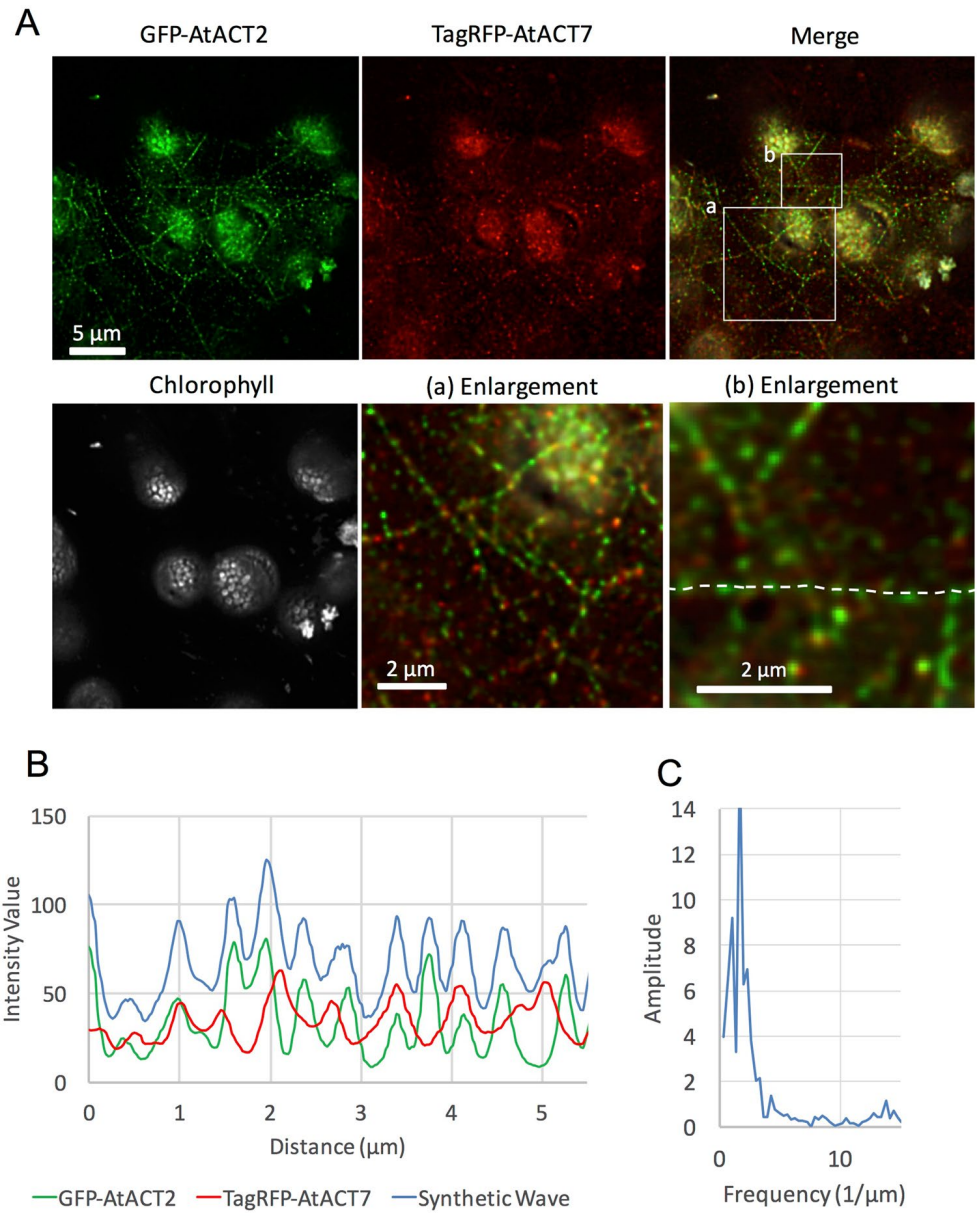
Distribution of GFP-AtACT2 and TagRFP-AtACT7 in spongy mesophyll cells from *N*. *benthamiana* leaves. (**A**) Deconvoluted optical section images of a representative spongy mesophyll cell transiently co-expressing GFP-AtACT2 (top left) and TagRFP-AtACT7 (top middle). White signals show auto-fluorescence from chloroplasts (bottom left). The merged image of GFP-AtACT2 (green), TagRFP-AtACT7 (red) and chlorophyll auto-fluorescence (gray) is shown on the top right. White boxes, a and b, indicate areas enlarged on the bottom middle and bottom right, respectively. GFP-AtACT2 and TagRFP-AtACT7 were not uniformly distributed in the filament and formed segregated patches with more or less regular spacing along the filaments. (**B**) The fluorescent intensity value profiles along the white dotted line shown in (**A**) at the bottom right ((b) enlargement). The individual profiles of GFP-AtACT2 and TagRFP-AtACT7 were from the green channel image and the red channel image, respectively, and the blue line shows the sum of the profiles of GFP-AtACT2 and TagRFP-AtACT7, after adjusting the intensities of each channel so that the average of the two intensity profiles would be the same. (**C**) The periodicity of the summed fluorescence intensity of (**B**) was calculated by fast Fourier transformation. The peak frequency was 2.25 µm^−1^, indicating that the periodicity was 0.44 µm.

To observe spongy mesophyll cells, we peeled off the thin epidermis from the abaxial side of a leaf (Supplemental Fig. 2). In the cytoplasm of spongy mesophyll cells, GFP-AtACT2 and TagRFP-AtACT7 were incorporated into the same fine filaments that extended in various directions, but the two fluorescent actins were not uniformly distributed in the filaments and formed segregated patches (Fig. 3A) with more or less regular intervals along the filaments (Fig. 3B). We speculate that these thin filaments were single filaments rather than bundles. This is because if these filaments were bundles, we have to assume that patches of fluorescent actin would have formed in a coordinated manner in different actin filaments within the bundle. The interval of the patches of GFP-AtACT2 and TagRFP-AtACT7 was approximately 0.44 µm according to a fast Fourier transform analysis (Fig. 3C). The gaps between patches of GFP-AtACT2 and TagRFP-AtACT7 along apparently contiguous filaments were perhaps occupied by endogenous *N*. *benthamiana* actin (Supplemental Fig. 3). These results suggest that AtACT2 and AtACT7 have the capacity co-polymerize into single filaments in *N*. *benthamiana* mesophyll cells, but are segregated in their distribution along the filament backbone.

**Figure 4:**
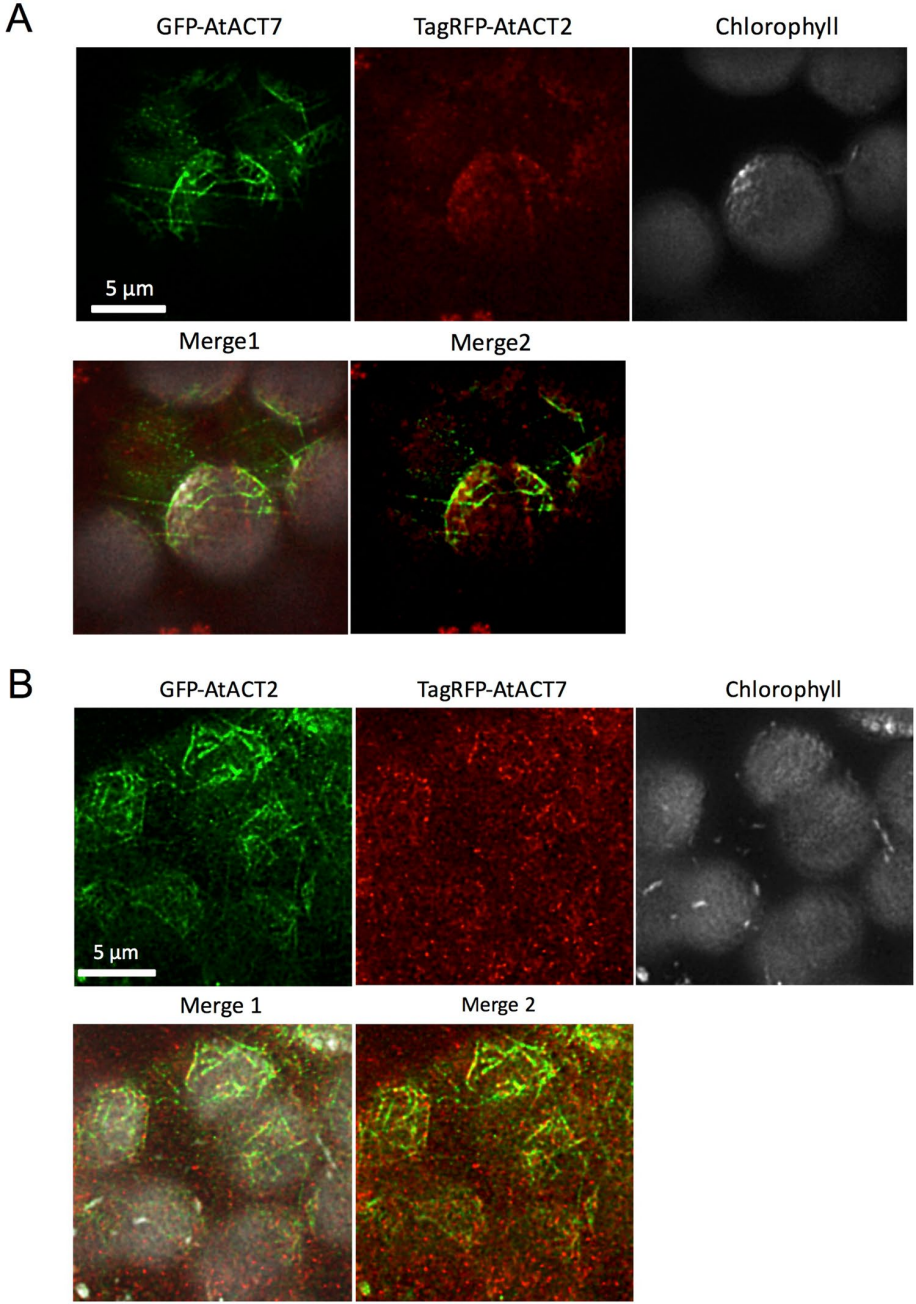
Distribution of GFP-AtACT2 and TagRFP-AtACT7 around chloroplasts in spongy mesophyll cells from *N*. *benthamiana* leaves. (**A**) Deconvoluted optical section images around chloroplasts in a representative spongy mesophyll cell transiently co-expressing GFP-AtACT7 (top left) and TagRFP-AtACT2 (top middle). Gray image shows auto-fluorescence from chloroplasts (top right). The merged image of GFP-AtACT7 (green), TagRFP-AtACT2 (red) and chlorophyll auto-fluorescence (white) is shown on the bottom left (merge 1). To make it easier to compare the fluorescence of GFP and TagRFP, the merged image of GFP-AtACT7 (green) and TagRFP-AtACT2 (red) is shown on the bottom center (merge 2). (**B**) Reciprocal experiment with GFP-AtACT2 and TagRFP-AtACT7. Deconvoluted optical section images around chloroplasts transiently co-expressing GFP-AtACT2 (top left) and TagRFP-AtACT7 (top middle). Gray image shows auto-fluorescence from chloroplasts (top right). The merged image of GFP-AtACT2 (green), TagRFP-AtACT7 (red) and chlorophyll auto-fluorescence (gray) is shown on the bottom left (merge 1), and the merged image of GFP-AtACT2 (green) and TagRFP-AtACT7 (red) is shown on the bottom center (merge 2).

We also tested whether GFP-actin is incorporated into filaments around chloroplasts. Antibodies specific for plant actin, phalloidin and GFP-mTalin1 stained specific subsets of actin filaments associated with chloroplasts^33–38^. Those actin filaments, now named chloroplast-actin (or cp-actin) filaments, are involved in chloroplast photorelocation movements^36^. Filaments of both GFP-AtACT2 and GFP-AtACT7 were found on the surface of chloroplasts (Fig. 4). The observed filament structures were similar to previously reported structures^34,36,37^, suggesting that both vegetative actin isoforms are capable of forming cp-actin filaments. Due to weak TagRFP fluorescence (Fig. 4), however, it was difficult to compare the distribution of AtACT2 and AtACT7 on chloroplast surfaces.

### Distribution of AtACT2 and AtACT7 in leaf epidermal cells

**Figure 5:**
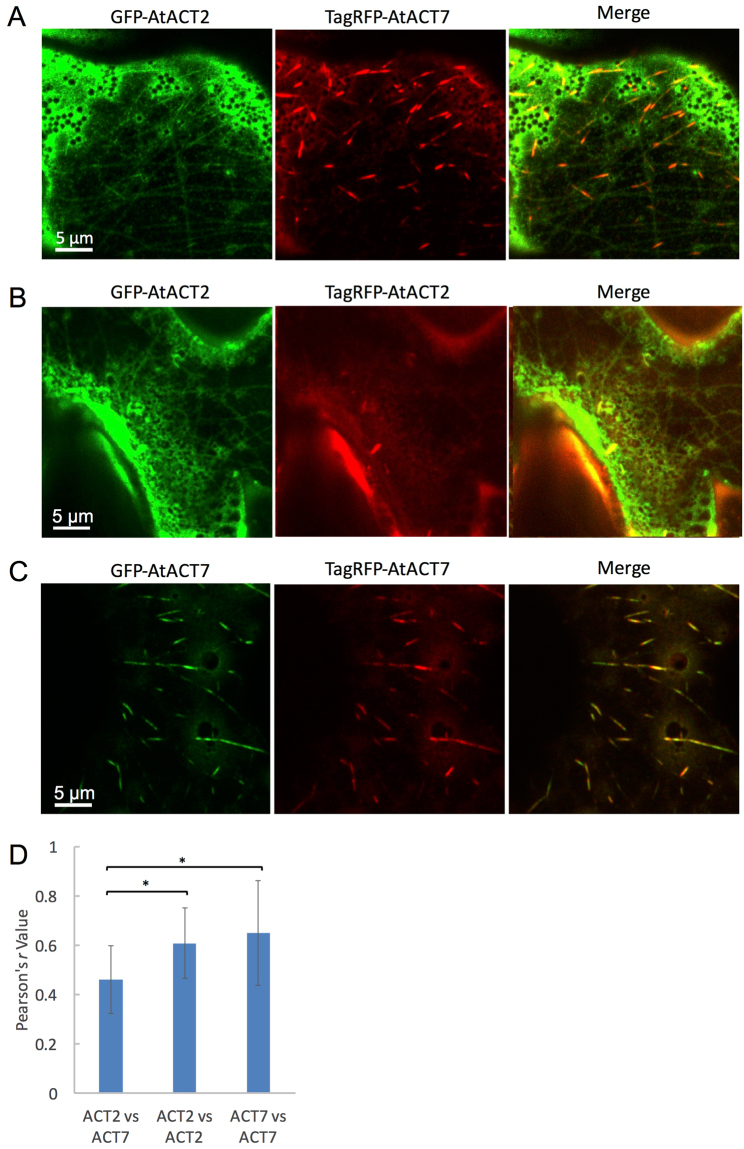
Distribution of AtACT2 and AtACT7 in *N*. *benthamiana* leaf epidermal cells. (**A**) Z-series projections of a representative leaf epidermal cell transiently co-expressing GFP-AtACT2 (left) and TagRFP-AtACT7 (middle). The merged image is shown on the right. (**B**) Z-series projections of another leaf epidermal cell transiently co-expressing GFP-AtACT2 (left) and TagRFP-AtACT2 (middle). The merged image is shown on the right. (**C**) Z-series projection of a leaf epidermal cell transiently co-expressing GFP-AtACT7 (left) and TagRFP-AtACT7 (middle). The merged image is shown on the right. (**D**) Comparison of Pearson’s correlation co-efficient (*r* value) between GFP and TagRFP fluorescence fused with individual actin isoforms. These values were measured in more than 15 regions of 10 × 10 µm^2^ from >5 cells each. Each bar shows the mean ± standard deviation. ^*^Indicates significant difference at p < 0.01 (Student’s *t*-test).

To test whether AtACT2 and AtACT7 are incorporated into the same filaments in other cell types, we compared their distribution in leaf epidermal cells. Surprisingly, in cells co-expressing GFP-AtACT2 and TagRFP-AtACT7, these two isoforms were incorporated into completely different types of filament arrays. GFP-AtACT2 was incorporated into filaments that were thinner and longer than those containing TagRFP-AtACT7. We speculate that the thinner filamentous structures containing GFP-AtACT2 are isolated individual filaments or bundles of a small number of filaments. In contrast, TagRFP-AtACT7 was mainly found in thick bundles that were localized along filaments containing GFP-AtACT2 (Fig. 5A). To confirm that the different localization of AtACT2 and AtACT7 was not caused by artifacts of the different fluorescent proteins (GFP and TagRFP), we expressed the same actin isoforms fused with the two fluorescent proteins, such as GFP-AtACT2 and TagRFP-AtACT2 (Fig. 5B). In cells co-expressing GFP-AtACT2 and TagRFP-AtACT2, thin filaments containing GFP-AtACT2 were observed, but TagRFP-AtACT2 filaments were hardly visible, presumably due to the dim fluorescence of TagRFP. In cells co-expressing GFP-AtACT7 and TagRFP-AtACT7, bundled filaments were observed in both color channels, as expected (Fig. 5C). Time-lapse imaging demonstrated that thin filaments of GFP-AtACT2 and thick bundles of GFP-AtACT7 are both dynamic (Supplemental Fig. 4).

We compared the degree of co-localization of the two fluorescent filament images quantitatively using Pearson’s correlation coefficient. A Pearson’s *r* value of + 1 indicates a perfect correlation, whereas a value of 0 implies no correlation. The *r* value for the combination of GFP-AtACT2 and TagRFP-AtACT7 was significantly lower than that for the same actin isoforms (Fig. 5D). This result suggests that the different isoforms display a distinct localization in the leaf epidermal cells. However, the formation of different types of AtACT2 and AtACT7 filaments was not observed in fixed cells presumably due to some artefacts of the fixation procedure, although their fluorescent intensities were uneven along the filaments (Supplemental Fig. 5).

### Distribution of *Arabidopsis* vegetative actin isoforms and generic actin filament probes in leaf epidermal cells

In epidermal cells, actin filaments have been visualized by various probes such as phalloidin or indirect fluorescent-fusion protein probes. Interestingly, several patterns of actin filaments have been observed in leaf epidermal cells^26,39–41^. Thus, we attempted to examine whether these fluorescent probes uniformly label all actin isoforms in leaf epidermal cells. To achieve this, we employed mTalin1 and Lifeact as commonly-used actin filament probes.

**Figure 6:**
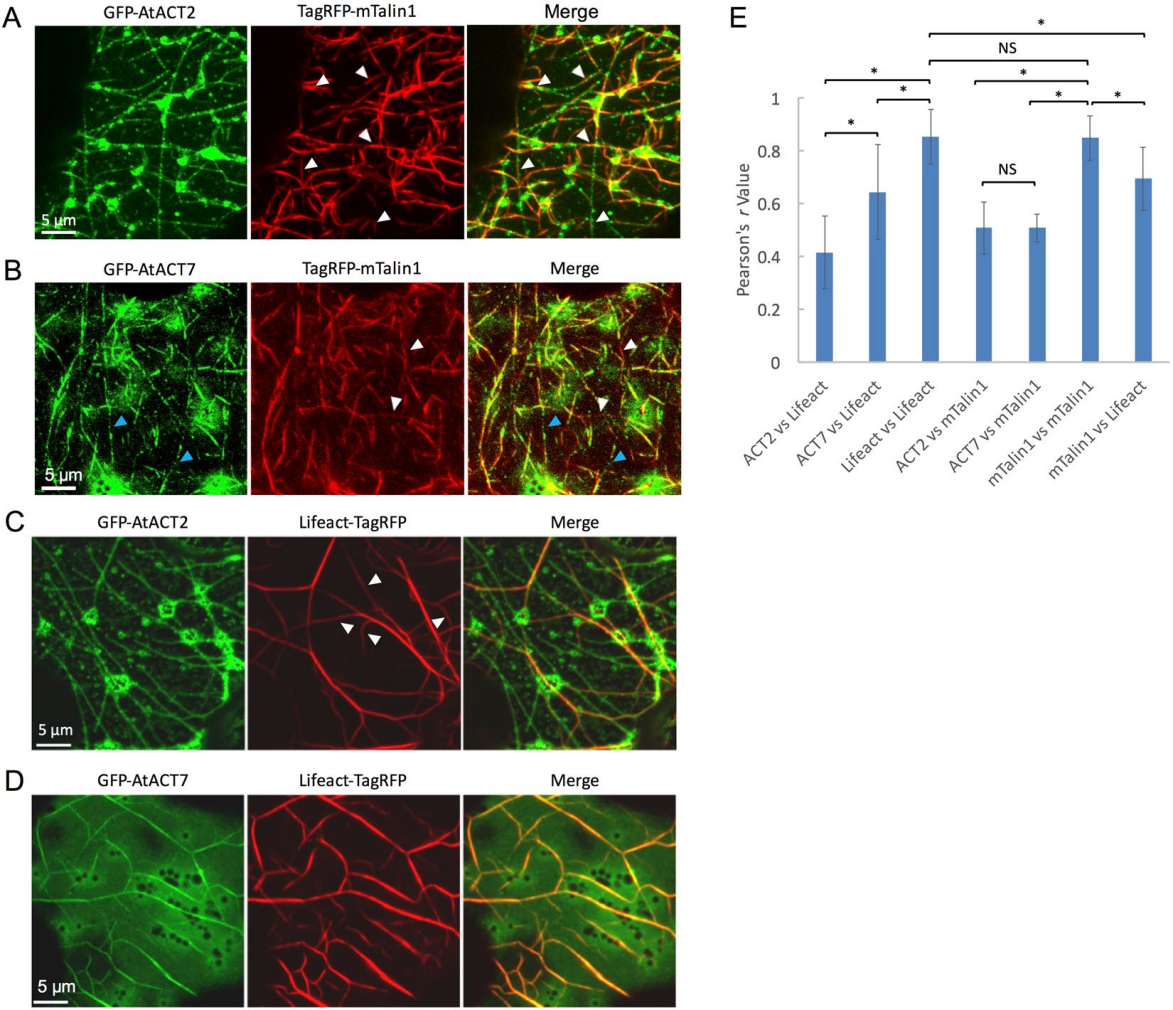
Distribution of *Arabidopsis* vegetative actin isoforms, AtACT2 and AtACT7, and generic actin filaments probes, mTalin1 and Lifeact, in *N*. *benthamiana* leaf epidermal cells. (**A**) Z-series projection of a representative leaf epidermal cell transiently co-expressing GFP-AtACT2 (left) and TagRFP-mTalin1 (middle). The merged image is shown on the right. White arrowheads indicate filaments labeled with mTalin1 localized along long AtACT2 filaments. (**B**) Z-series projection of an epidermal cell transiently co-expressing GFP-AtACT7 (left) and TagRFP-mTalin1 (middle). The merged image is shown on the right. Blue and white arrowheads indicate filaments observed only in either the green or red channel. (**C**) Z-series projection of an epidermal cell transiently co-expressing GFP-AtACT2 (left) and Lifeact-TagRFP (middle). The merged image is shown on the right. White arrowheads indicate filaments containing almost no GFP-AtACT2. (**D**) Z-series projection of an epidermal cell transiently co-expressing GFP-AtACT7 (left) and Lifeact-TagRFP (middle). The merged image is shown on the right. (**E**) Comparison of Pearson’s correlation co-efficient (*r* value) between GFP and TagRFP fluorescence fused with individual proteins. These values were measured in more than 30 regions of 10 × 10 µm^2^ from > 10 cells each. Each bar shows the mean ± standard deviation. ^*^Indicates significant difference at p < 0.01 (Student’s *t*-test). NS indicates no significant difference.

GFP-mTalin1 labeled a dense actin filament meshwork in protoplasts (Fig. 1C) and leaf epidermal cells (Supplemental Fig. 6). Unexpectedly, in cells co-expressing TagRFP-mTalin1 and GFP-AtACT2 or GFP-AtACT7, the fluorescence of mTalin1 did not completely overlap with that of individual actin isoforms (Fig. 6A,B). It seems that relatively short filaments which were labeled with mTalin1 localized along long AtACT2 filaments (Fig. 6A, arrowheads). In contrast, filamentous structures labeled by mTalin1 and those of GFP-AtACT7 were very similar, although some filament sections were labeled by only one of the two fluorescent probes (Fig. 6B, arrowheads). In the Pearson’s correlation analysis, the *r* value for the combinations of GFP-AtACT2 or GFP-AtACT7 and TagRFP-mTalin1 were significantly lower than that of GFP-mTalin1 and TagRFP-mTalin1 (Fig. 6E).

Next, we compared the distribution of GFP-actin isoforms and Lifeact-TagRFP. Lifeact is composed of only 17 amino acid residues, and is derived from the yeast ABP, Abp140p^42^. Lifeact has been widely employed as an actin filament probe not only in plants^27^ but also in other organisms including animal cultured cells^42^, yeast^42^ and *Dictyostelium*^43^. The filamentous structures labeled with Lifeact were thick and sparse relative to filaments labeled with mTalin1 in protoplasts (Fig. 1C) and leaf epidermal cells (Supplemental Fig. 6). Surprisingly, in cells co-expressing GFP-AtACT2 and Lifeact-TagRFP, many thinner filaments containing GFP-AtACT2 were not stained by Lifeact-TagRFP, while some filaments labeled with Lifeact-TagRFP hardly contained GFP-AtACT2 (Fig. 6C, arrowheads). In contrast to GFP-AtACT2, GFP-AtACT7 showed thick bundled filaments with a high affinity to Lifeact-TagRFP (Fig. 6D). In agreement with these observations, the Pearson’s correlation coefficient between GFP-AtACT2 and Lifeact-TagRFP was significantly lower than that between GFP-AtACT7 and Lifeact-TagRFP (Fig. 6E).

Finally, we compared the staining patterns in cells co-expressing mTalin1 and Lifeact. We predicted that mTalin1 and Lifeact would separately label different types of filaments because filamentous structures labeled with either mTalin1 or Lifeact were distinct (Supplemental Fig. 6). Contrary to this expectation, Lifeact and mTalin1 localized along the same actin filaments, although the labeling intensities were uneven along the filaments (Supplemental Fig. 7). In agreement with this observation, the Pearson’s correlation coefficient between mTalin1 and Lifeact was statistically significantly but subtly lower than between the combination of the same probes, mTalin1 and mTalin1 or Lifeact and Lifeact (Fig. 6E). Interestingly, actin filaments stained with mTalin1 and Lifeact could be distinguished into two types according to organization of the filament arrays. Some cells showed thin and dense filament arrays, namely, the mTalin1 type (Supplemental Fig. 7, upper panels), whereas others showed thick and sparse filament arrays, namely, the Lifeact type (Supplemental Fig. 7, lower panels). Moreover, the filamentous pattern of GFP-AtACT7 co-expressing TagRFP-mTalin1 was also obviously different from GFP-AtACT7 filaments in cells co-expressing Lifeact-TagRFP (Fig. 6B,D). This result suggests that expression of actin filament-binding probes substantially perturbs the structure of endogenous and expressed actin isoforms.

### Binding of Lifeact-GFP to vegetative actin isoforms *in vitro*

The mTalin1 and Lifeact probes differentially decorated AtACT2- and AtACT7-containing filaments in epidermal cells (Fig. 6). Specifically, Lifeact-TagRFP scarcely labeled a number of filaments containing GFP-AtACT2, whereas Lifeact-TagRFP colocalized well with GFP-AtACT7 (Fig. 6C,D). To uncover the reason for these differences, we attempted to examine whether Lifeact-GFP binds to purified AtACT2 filaments *in vitro* by fluorescence microscopic observation and a cosedimentation assay. If some of the amino acid substitutions in AtACT2 disrupt the binding site for Lifeact, then binding of Lifeact-GFP to purified AtACT2 filaments should not be observed *in vitro* as well.

**Figure 7:**
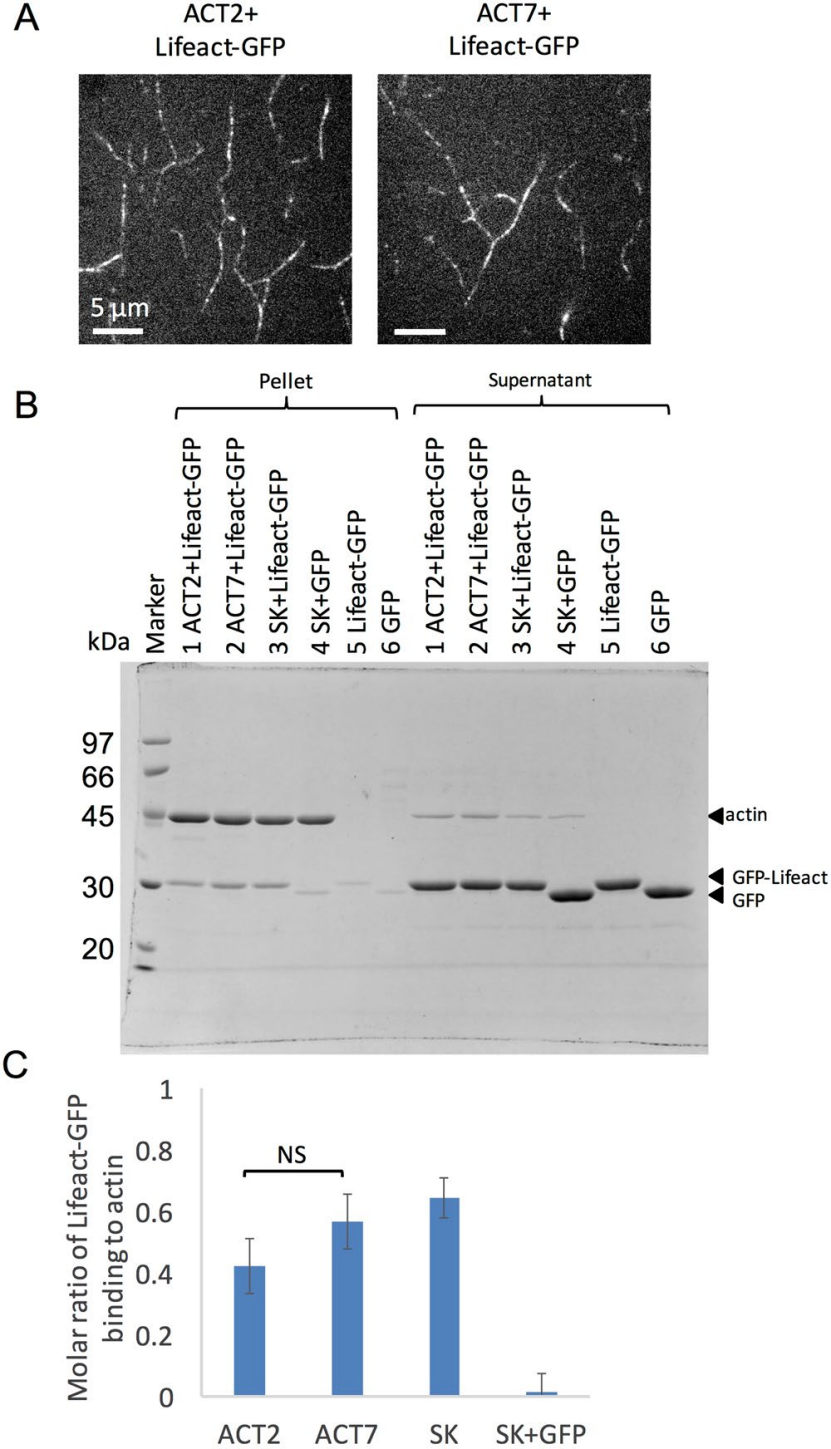
Analysis of Lifeact-GFP binding to AtACT2 and AtACT7 filaments *in vitro*. (**A**) Fluorescent images of Lifeact-GFP after incubation with each unlabeled actin isoform filaments. Unlabeled actin filaments were immobilized on an amino silane-coated glass chamber. The binding reaction was performed in F-buffer (10 mM HEPES (pH 7.4), 50 mM KCl, 2 mM MgCl_2_, 1 mM ATP, 10 mM DTT) containing 1 mg/ml BSA and 4 µM Lifeact-GFP. GFP fluorescence was localized linearly in both mixtures of unlabeled AtACT2 and AtACT7 filaments. (**B**) Cosedimentation of 8 µM Lifeact-GFP with 4 µM individual actin isoform filaments as follows. Sample 1: 4 µM AtACT2 + 8 µM Lifeact-GFP, Sample 2: 4 µM AtACT7 + 8 µM Lifeact-GFP, Sample 3: 4 µM skeletal muscle actin (SK) + 8 µM Lifeact-GFP, Sample 4: 4 µM skeletal muscle actin (SK) + 8 µM GFP, Sample 5: 8 µM Lifeact-GFP and Sample 6: 8 µM GFP. Supernatant and pellet fractions after ultra-centrifugation were separately loaded to 12% SDS-PAGE. (**C**) The molar ratio of Lifeact-GFP binding to actin filaments was calculated by dividing the amount of Lifeact-GFP in the pellet by the amount of actin filaments in the pellet (mol/mol). The amount of Lifeact-GFP in the control pellet, representing non-specific precipitation or binding to the wall of the tube (Lane 5 in the Pellet) was subtracted from the amounts of Lifeact-GFP in each pellet. Each bar shows the mean ± standard deviation (n = 4). NS indicates no significant difference at p > 0.05 (Student’s *t*-test).

To detect binding of Lifeact-GFP to actin filaments using fluorescence microscopy, actin filaments were immobilized to a glass surface modified with amino-silane^44^. After incubation with 4 µM Lifeact-GFP, GFP fluorescence was arranged linearly when either AtACT2 filaments or AtACT7 filaments were used (Fig. 7A), demonstrating that Lifeact-GFP binds to both purified AtACT2 and AtACT7 filaments *in vitro*. To compare the extent of Lifeact-GFP binding more quantitatively, we performed a cosedimentation assay of purified individual actin isoforms and Lifeact-GFP. When Lifeact-GFP was mixed with filaments of either isoform, the amount of Lifeact-GFP in the pellet was much larger than that in the absence of actin filaments (Fig. 7B). The molar ratio of Lifeact-GFP bound to AtACT2 was slightly lower than that to AtACT7, but the difference was not significant (Fig. 7B,C; student’s *t*-test; p > 0.05). These results demonstrate that Lifeact-GFP is capable of binding to purified AtACT2 filaments *in vitro*.

## Discussion

GFP-actin technology, which has been successfully used in animal and yeast cells^21,22,45^, is able to distinguish between actin isoforms. However, there has been few reports of visualizing GFP-actin in plant cells, such as enrichment of actin-GFP around the nuclei and near the tip and cell wall in *N*. *tabacum* BY2 suspension cells^23^, and bundle-like patches of actin-GFP on mitochondria in *N*. *tabacum* protoplasts^24^. In this study, therefore, we developed GFP-actin constructs for use in plant cells. Specifically, we examined the linker length and the position of GFP as orthodox practices of a fluorescent-fusion protein. As a result, we succeeded in observing filaments containing GFP-actin, in which actin is fused to the C-terminus of GFP with a long linker (Fig. 1). GFP-actin expressed in budding yeast was able to be incorporated into actin patches but was unable to localize in contractile rings^46^, suggesting that the N-terminal GFP moiety interferes with polymerization catalyzed by Cdc12p formin that is specifically responsible for contractile ring formation. Likewise, it may be that major actin polymerization factors in plant cells are inhibited by the N-terminal GFP moiety too closely positioned to the actin moiety. Paradoxically, by an unknown mechanism, fusion of GFP to the N-terminus of actin through a long linker increases the amount of filaments *in vivo* in both plant and animal cells^31^. Thus, the reason why no long filaments were observed in previous studies using actin-GFP in plant cells^23,24^ is probably fusion of GFP to the C-terminus of actin. By using the actin fluorescent-fusion protein, we revealed that the distribution patterns of different actin isoforms were different depending on the cell types of the leaf. Specifically, in leaf epidermal cells, GFP-AtACT2 and TagRFP-AtACT7 were incorporated into different types of filaments (Fig. 5A), suggesting that these actin isoforms are functionally distinct.

In mesophyll cells, actin filaments were localized in at least two different regions, in the cytoplasm and around chloroplasts. In cytoplasm, GFP-AtACT2 and TagRFP-AtACT7 co-polymerized in a segregated manner (Fig. 3A). One function of cytoplasmic actin filaments is to provide tracks for myosin-dependent transport of organelles and vesicles. Actin filament assembly can also power movement of membrane-bound compartments, for example, the chloroplast. Notably, both AtACT2 and AtACT7 were found on the surface of chloroplasts (Fig. 4), and could work as cp-actin filaments^36^. The functions of actin filament arrays in different regions of a cell are likely distinct, but given their co-existence at several locations, AtACT2 and AtACT7 probably work cooperatively and share overlapping functions. In unicellular eukaryotes such as *Dictyostelium* and in mammalian cells, actin is referred to as “a universal force provider”^32^. In these organisms, actin fulfills complex and various functions through interactions with a variety of ABPs and/or by undergoing cooperative conformational changes that alter affinities for different ABPs^44^. Genetic complementation experiments by Kandasamy *et al*. demonstrated that over-expression of only AtACT8 (note that AtACT8 differs from AtACT2 by only one amino acid residue) is sufficient for normal development of *Arabidopsis* in the absence of the major vegetative actin isoforms, AtACT2 and AtACT7^10^. This result suggests functional redundancy among the vegetative actin isoforms, but does not necessarily rule out the possibility of specific functions for actin isoforms. For instance, complete complementation of the AtACT2/AtACT7 knock-out phenotype required AtACT8 to be expressed at about twice the expression level of total actin in wild type plants^10^, suggesting that the function of AtACT8 is incomplete when substituting as a single vegetative actin isoform. In addition, over-expression of AtACT7 did not fully rescue root hair growth in the absence of AtACT2 and/or AtACT8^10^. Moreover, there is a possibility that the subtle phenotypic difference among the wild-type and knock-out lines of actin isoforms could not be detected during a single generation. Over 200 million years have elapsed since AtACT7 and AtACT2/AtACT8 diverged from a common ancestor^6^. Thus, even if the growth advantage of having both ACT2/8 and ACT7 isoforms is relatively small, the accumulated selective advantage during 200 million years of evolution would be obviously huge. Finally, the knock-out lines of actin isoforms were grown and evaluated under greenhouse conditions^10^, but their phenotypes in the presence of various environmental or abiotic stresses such as drying, adverse temperature and light conditions, or biotic stresses, are unknown. Further studies will be necessary to reveal the physiological relevance of the segregated filaments and the relationship between various actin functions and actin isoforms.

In epidermal cells, AtACT2 and AtACT7 were incorporated into significantly different types of filaments (Fig. 5A). AtACT2 was incorporated into thinner filamentous structures, whereas AtACT7 was incorporated into thick bundles. It is expected that AtACT2 and AtACT7 perform specific functions in epidermal cells. Since epidermal cells are located on the outer most surface of the leaf, actin filaments in epidermal cells would contribute to a wider variety of functions, such as the capacity to respond to external stimuli, and may need more diverse filamentous structures. Interestingly, several studies demonstrated that actin is involved in plant innate immunity^47,48^. For example, infection by pathogenic bacteria promptly increased the density of actin filaments in epidermal cells^49^. Kang *et al*. demonstrated that HopW1, an effector protein delivered into host cells by *Pseudomonas syringae*, increases the growth of the pathogen on plant tissues, disrupts filaments of non-muscle actin, but not muscle actin *in vitro*, and decreases the abundance of actin filaments in epidermal cells of *Arabidopsis*^50^. Since *Arabidopsis* vegetative actin isoforms are more diverse than animal actin isoforms, it is suggested that HopW1 interacts with specific actin isoforms that are involved in innate immunity. Viewed in this light, in epidermal cells, it is interesting to speculate that specific types of filaments containing either AtACT2 or AtACT7 contribute to a response to an immune signal and/or other external signals. Further studies on the relationship between actin isoforms, ABPs, and effector proteins delivered by microbes are required to understand the physiological meaning of these different types of filaments.

Interestingly, generic actin filament probes, mTalin1 and Lifeact, differentially stained AtACT2- and AtACT7-containing filaments in epidermal cells (Fig. 6). However, *in vitro* binding analyses revealed that Lifeact-GFP binds to purified AtACT2 filaments similarly to AtACT7 filaments (Fig. 7), ruling out the possibility of AtACT2 having amino acid substitutions at the Lifeact binding site. The partial staining of actin filaments by Lifeact *in vivo* is probably caused by the competition of Lifeact with endogenous ABPs which prefer filaments containing AtACT2 over those containing AtACT7. A large number of ABPs are present at substantial concentrations in plant cells. For example, the molar ratio of ADF to total actin reaches 1:3 in *Arabidopsis* rosette leaves^51^. The fact that a high level of expression of Lifeact-GFP was required to label the actin structure called actin collard in pollen tube^52,53^ also supports competition between expressed Lifeact-GFP and endogenous ABP(s). Moreover, several studies suggested an actin isoform-dependent interaction with ABPs^14,54–56^. Thus, it is not surprising that specific endogenous ABPs occupy the binding sites for Lifeact-GFP along actin filaments. Furthermore, the affinity of certain ABPs, such as cofilin and profilin, varies with the nucleotide state of actin protomers^57,58^. Since the phosphate release rate was significantly different between AtACT2 and AtACT7 *in vitro*^14^, it is plausible that the nucleotide state of each actin isoform in filaments would be different in cells and this would introduce another source of differential affinity for ABPs between actin isoforms. With respect to the differential localization of actin probes using ABDs, it has also been reported that GFP fused to ABDs derived from fimbrin, plastin and talin in *Arabidopsis*^59^, and from filamin and α-actinin in *Dictyostelium* cells^60^ showed distinct intracellular localization patterns. In animal cells from various model organisms, actin filament probes, including Lifeact and those derived from ABDs of F-tractin and utrophin, also showed a biased distribution^61^.

Actin probes, mTalin1 and Lifeact, which have been widely used to visualize actin filaments in plant cells, do not label all intracellular actin filaments uniformly, questioning their validity as generic actin filament probes. In addition, the expression of mTalin1 and Lifeact apparently affected intracellular actin filament patterns in epidermal cells (Supplemental Fig. 6). Moreover, the co-expression of TagRFP-Lifeact or -mTalin1 caused different actin filament patterns of expressed GFP-AtACT7 (Fig. 6B,D). In line with this, several phenotypes associated with expression of generic actin probes have been reported^28^. Expression of GFP-mTalin1 reduced the growth of roots and hypocotyls, altered the morphology of the silique and reduced actin filament dynamics^26^. Transient expression of GFP-mTalin1 by an alcohol-inducible system affected root hair morphology and tip growth by causing a defect in actin organization^62^. *In vitro*, GFP-mTalin1 inhibited the actin depolymerizing activity of ADF^62^. Although Lifeact is a very small actin tag composed of only 17 amino acid residues, over-expression of Lifeact also affects the growth of root hairs and hypocotyls^29^, and plant size^63^. However, these Lifeact-induced phenotypes seemed to be milder than other actin probes^63,64^. Lifeact failed to associate with a number of AtACT2-containing filaments (Fig. 6C), thus plant cells presumably contain native actin filaments not affected by Lifeact. However, it must be noted that expression of GFP-actin also affected cell morphogenesis in various organisms^20,31,45,46^. Therefore, an appropriate actin probe needs to be selected depending on the purpose.

## Methods

### Plasmid Constructs

The expression vector for protoplasts was constructed using pUC18–35S-GFP-nosT^65^ (a kind gift from Dr. Sam-Geun Kong), which contained the GFP coding sequence between the cauliflower mosaic virus (CaMV) 35 S promoter and the NOS terminator (Supplemental Fig. 8). First, cDNA of either GFP or TagRFP was additionally inserted upstream of the original GFP sequence and downstream of the CaMV35S promoter between *Sal*I and *Nco*I sites with various lengths of a linker. The coding sequence of GFP was obtained from pUC18–35S-GFP-nosT by PCR using the sense primer 5′-CTGgtcgacATGGTGAGCAAGGGC-3′ and the antisense primers 5′-CTAccatggAAGATCCCTTGTACAGCTCGTCC-3′ for the GSS short linker or 5′-CTAccatggAGGATCCAGATGAACCAGAAGAACCCTTGTACAGCTCGTCC-3′ for the 3XGSS for the intermediate linker. The coding sequence of GFP having the long linker, 6XGSS, was obtained from pUC18-GFP-3XGSS-GFP-nosT by PCR using the sense primer 5′-CTGgtcgacATGGTGAGCAAGGGC-3′ and the antisense primer 5′-CTAccatggATGATCCTGATGACCCGGAAGATCCAGAGGATCCAGATGAACCAG-3′. Lower case letters indicate sequences added to generate recognition sites for restriction enzymes and underlined sequences indicate the coding sequences of the linker. cDNA of TagRFP contained in Gateway TagRFP-AS-N vector was obtained from Evrogen (Moscow, Russia). The coding sequence of TagRFP was obtained from TagRFP-AS-N-vector by PCR using the sense primer 5′-CTGgtcgacATGGTTTCTAAGG-3′ and the antisense primer 5′-CTAccatggAGGATCCAGATGAACCAGAAGAACCGTTCAATTTGTGACCTAGC-3′ for the 3XGSS intermediate linker. The linker of pUC18-TagRFP-3XGSS-GFP-nosT was elongated using the sense primer 5′-CTGgtcgacATGGTTTCTAAGG-3′ and the antisense primer 5′-CTAccatggATGATCCTGATGACCCGGAAGATCCAGAGGATCCAGATGAACCAG-3′. Note that these sense primers have a *Sal*I site at the 5′ end while the antisense primers have an *Nco*I site at the 5′ end. To fuse actin to the C-terminus of a fluorescent protein, the original GFP coding sequence between *Nco*I and *Not*I sites in the pUC18 vector was replaced with cDNA of AtACT2 or AtACT7. Each actin cDNA was obtained from pTIKLART-ACT15P-ACT2 or pTIKLART-ACT15P-ACT7^14^ by PCR. Primer sets used for this cloning procedure are as follows. AtACT2, 5′-CTGccatggCTGAGGCTGAT-3′ and 5′-CTAgcggccgcTTAGAAACATTTTCTGTGAAC-3′; AtACT7, 5′-CTGccatggCCGATGGTGA-3′ and 5′-CTAgcggccgcTTAGAAGCATTTCCTGTGA-3′. Note that the sense primers have an *Nco*I site at the 5′ end while the antisense primers have a *Not*I site at the 5′ end.

The cDNA of GFP-mTalin1 was a kind gift from Dr. Sam-Geun Kong. The coding sequence of mTalin1 was obtained from the cDNA of GFP-mTalin1 by PCR using the sense primer 5′-CTGccatggGAATCCTAGAAGCTGCCA-3′ and the antisense primer 5′-CTAgcggccgcTTAGTGCTCGTCTCGA-3′. Note that the sense primer has an *Nco*I site at the 5′ end while the antisense primer has a *Not*I site at the 5′ end. The coding sequence of mTalin1 was inserted between *Nco*I and *Not*I sites in pUC18-GFP-6XGSS-ACT7 or pUC18-TagRFP-6XGSS-ACT7 by replacing the AtACT7 sequence.

The cDNA of Lifeact-GFP was obtained from Lifeact/pEGFP-N1^31^ by PCR using the sense primer 5′-CTGgtcgacATGGGTGTCGCAGAT-3′ and the antisense primer 5′-CTAgcggccgcTTACTTGTACAGCTCGTC-3′. Note that the sense primer has a *Sal*I site at the 5′ end while the antisense primer has a *Not*I site at the 5′ end. The coding sequence of Lifeact-GFP was inserted between *Sal*I and *Not*I sites of the pUC18 vector. To replace the coding sequence of GFP fused with the coding sequence of Lifeact by the cDNA of TagRFP, the cDNA of TagRFP was amplified by PCR using the sense primer 5′-CTGccatggTTTCTAAGGGTGAAG-3′ and the antisense primer 5′-CTAgcggccgcTCAGTTCAATTTGTGACCT-3′. Note that the sense primer has an *Nco*I site at the 5′ end while the antisense primer has a *Not*I site at the 5′ end. The cDNA of TagRFP was inserted between *Nco*I and *Not*I sites in the rear of the Lifeact coding sequence.

The expression vector pRI 101-AN for expression in *N*. *benthamiana* leaves was obtained from Takara (Shiga, Japan). The expression vector contains a 5′ untranslated region derived from Alcohol Dehydrogenase of *A*. *thaliana* downstream of the CaMV 35S promoter. For cloning, a *Not*I site was inserted downstream of the *Sal*I site. Each of the coding sequences of GFP-actin, TagRFP-actin, GFP-mTalin1, TagRFP-mTalin1, Lifeact-GFP or Lifeact-TagRFP gene contained in the pUC18-derived vector were cloned between *Sal*I and *Not*I sites in the modified pRI 101-AN vector.

To purify Lifeact-GFP and GFP, coding sequences of Lifeact-GFP and GFP were amplified from Lifeact/pEGFP-N1 by PCR using the following primer sets. Lifeact-GFP: 5′-CTGggtaccATGGGTGTCGCAGATTT-3′ and 5′-CTGtctagaTTACTTGTACAGCTCGTCC-3′; GFP: 5′-ATCgggtaccATGGTGAGCAAGGGC-3′ and 5′- CTGtctagaTTACTTGTACAGCTCGTCC -3′. Note that the sense primers have a *Kpn*I site at the 5′ end while the antisense primers have an *Xba*I site at the 5′ end. Each coding sequence of Lifeact-GFP and GFP was cloned into the pColdTEV expression vector^14^.

To purify AtACT2 and AtACT7, coding sequences of AtACT2 and AtACT7 fused with thymosin β-His-tag were amplified from pTIKLART-ACT15P-ACT2 or pTIKLART-ACT15P-ACT7 and inserted into pFastBac1 (Invitrogen, CA, USA), which is a high-level protein expression vector using the Bac-to-Bac baculovirus system in insect cells.

### Preparation of protoplasts and *N. benthamiana* plants

The established *Arabidopsis* cell line, T87, was obtained from the RIKEN Plant Cell Bank. T87 cells were cultured in Jouanneau and Péaud-Lenoël medium^66,67^ with gentle suspension (120 rpm) at 22 °C under continuous white light^68^. *N*. *benthamiana* seeds were germinated and grown in soil under a 16-hr photoperiod at 26 °C. Plants 3–4 weeks old were used for infiltration experiments.

### Transformation

Transient gene expression in protoplasts was performed using the polyethylene glycol (PEG)-calcium transfection method^69^.

For *Agrobacterium* infiltration, individual pRI101 plasmids were transformed into *Agrobacterium tumefaciens* GV3101. Transient gene expression in *N*. *benthamiana* leaves was performed using the *Agrobacterium* infiltration method originally developed by Goodin *et al*.^70^. Transformed *Agrobacterium* cells were cultured in LB medium containing 50 µg/ml rifampicin (Wako, Osaka, Japan), 20 µg/ml gentamicin (Wako) and 50 µg/ml kanamycin (Wako) at 28 °C for 24–36 hr. Cells were harvested and washed with MES buffer (10 mM MES (pH 5.6), 10 mM MgCl_2_, 100 µM acetosyringone) and adjusted to an OD_600_ of 1.0 with MES buffer. For co-infiltration of *Agrobacterium* carrying different plasmids such as GFP and TagRFP, the mixing volume ratio between GFP and TagRFP was 1:2. After incubation for 2–3 hr at room temperature, the *Agrobacterium* suspension was slowly infiltrated into the leaf abaxial surface using a 1 ml syringe. After incubation for 40–48 hr in a growth chamber, the infiltrated leaves were observed.

### Microscopic observation

For confocal fluorescence imaging of protoplasts, we used an inverted microscope (IX71; Olympus, Tokyo, Japan) equipped with a spinning disk confocal scanning unit (CSU10; Yokogawa, Tokyo, Japan), a cooled CCD camera (ORCA-ER; Hamamatsu Photonics, Shizuoka, Japan) and a Plan Apo 60 × /1.40 NA oil-immersion objective (Olympus). Illumination was from a 488-nm laser through a single band pass filter (FF03-525/50-25; OPTO-LINE, Tokyo, Japan) to reduce the fluorescence from chlorophylls. Before observation, the protoplasts were transferred to a glass-bottom dish (Matsunami, Osaka, Japan).

For confocal fluorescence imaging of *N*. *benthamiana* leaves, we used an inverted microscope (Eclipse Ti-E; Nikon, Tokyo, Japan) equipped with a laser scanning confocal microscope system (A1R; Nikon), a GaAsP detector (Nikon) and an oil-immersion objective (Apo 60 × /1.4 NA Oil λs, Nikon). Optically sectioned images in Figs 3 and 4 were deconvoluved by an imaging software, NIS-Elements (version 4.51, Nikon). All confocal images of Z-projection and the gray value profile were processed by ImageJ software. Before observation, a transformed leaf was de-aired using a 10 ml syringe in water. The leaf was held in a thin chamber, covered with a coverslip (No. 1 S 24 mm × 24 mm; Matsunami) at the top and sealed with wax (the mixture of bees wax (Wako) and lanoline (Wako) in the weight ratio of 1:1). The thin chamber was made of a glass slide and two parallel glass strips of coverslips (C050701 0.13-0.17 mm thickness; Matsunami) glued by ultraviolet curable resin (Norland optical adhesive 61; Norland Products, NJ, USA). When observing leaf mesophyll cells, the epidermis of the leaf was peeled off using tweezers with a sharp point. The Pearson’s correlation co-efficient (*r* value) was calculated using coloc-2 plugin included in Fiji software in 10 × 10 µm^2^ ROIs containing filaments.

A fluorescence microscope (IX-70, Olympus) equipped with a UPlan-Apo 100 × /1.35 NA oil-immersion objective (Olympus) and a sCMOS camera (ORCA-Flash 2.8, Hamamatsu Photonics) was used to observe the binding of Lifeact-GFP to actin filaments.

### Protein purification

AtACT2 and AtACT7 were expressed in Sf9 insect cells (Novagen, WI, USA) by infection with recombinant baculoviruses carrying AtACT2-thymosin-His or AtACT2-thymosin-His. The infected cells were cultured at 28 °C in 175 cm^2^ flasks and harvested after 3 days. Insect cells were harvested and AtACT2 and AtACT7 were purified as described previously^14^.

Rabbit skeletal muscle actin was prepared by a previously described method^71^.

Lifeact-GFP was expressed as a fusion with 6XHis and the TEV cleavage site on the N-terminus in *E*. *coli* strain Resetta (DE3) (Novagen) and purified using Ni-Sepharose 6 Fast Flow (GE Healthcare, Tokyo, Japan) affinity chromatography and eluted by elution buffer (10 mM HEPES (pH 7.4), 200 mM imidazole (pH 7.4), 7 mM β-mercaptoethanol). After the addition of His-TEV protease to the eluted His-TEV-Lifeact-GFP fusion protein at a 1/30 (w/w) ratio, the solution was dialyzed against dialysis buffer (10 mM HEPES (pH 7.4), 50 mM KCl, 0.2 mM DTT, 0.01% NaN_3_) overnight at 4 °C. His-tag and His-TEV protease were removed by passing through the Ni-Sepharose. Finally, Lifeact-GFP was concentrated using a centrifugal filter unit (Amicon Ultra filters 10 K; Millipore, MA, USA). Purified Lifeact-GFP was snap-frozen and stored at −80 °C. GFP was expressed and purified using the same method as Lifeact-GFP.

### Observation of Lifeact-GFP binding to actin filaments

Cover slips coated with positively charged silane were prepared by dipping cover slips in 1/20,000 diluted 3-aminopropyltrimethoxysilane (T1255; Tokyo Chemical Industry, Tokyo, Japan) in Milli-Q water for 5 min at room temperature. These cover slips were then rinsed with Milli-Q water three times and dried at room temperature. A flow chamber was made using double-sided tape and a glass slide. Cover slips coated with amino silane were used within 2 days after silanization.

AtACT2 and AtACT7 were polymerized at a concentration of 3 µM in F-buffer (10 mM HEPES (pH 7.4), 50 mM KCl, 2 mM MgCl_2_, 1 mM ATP, 10 mM DTT) on ice for 2 hr. Individual filamentous actin was diluted to 40-80 nM in F-buffer and was introduced into a flow chamber with an amino silane-modified surface. After incubation for 3 min, the chamber was washed with F-buffer containing 1 mg/ml bovine serum albumin (BSA; Wako) to remove unbound actin filaments. Lifeact-GFP diluted to 4 µM in F-buffer containing 1 mg/ml BSA was then introduced into the flow chamber. After 5 min, the chamber was washed again with F-buffer containing 1 mg/ml BSA and a fluorescence image of Lifeact-GFP was captured.

### Cosedimentation assay

AtACT2, AtACT7 and skeletal muscle actin were polymerized at 15 µM in F-buffer on ice for at least 1 hr. Individual actin isoforms and Lifeact-GFP were then mixed at a final concentration of 4 µM and 8 µM in F-buffer, respectively, and incubation was continued for 15 min at room temperature. After incubation, the mixtures were ultra-centrifuged at 286,000 × g for 10 min at 22 °C. The supernatants and pellets were applied to a 12% SDS-PAGE gel and loaded after dissolving in SDS-sample buffer and heating at 95 °C for 5 min. The amount of binding of Lifeact-GFP to actin was calculated using ImageJ.

The datasets generated and analysed during the current study are available from the corresponding author on reasonable request.

## Electronic supplementary material

Supplementary Information

## References

[1] Hussey PJ, Ketelaar T, Deeks MJ (2006). Control of the actin cytoskeleton in plant cell growth. Annu. Rev. Plant Biol..

[2] Volkmann D, Baluska F (1999). Actin cytoskeleton in plants: from transport networks to signaling networks. Microsc. Res. Tech..

[3] McCurdy DW, Kovar DR, Staiger CJ (2001). Actin and actin-binding proteins in higher plants. Protoplasma.

[4] Kawashima T (2014). Dynamic F-actin movement is essential for fertilization in Arabidopsis thaliana. Elife.

[5] Nick P, Han M-J, An G (2009). Auxin stimulates its own transport by shaping actin filaments. Plant Physiol..

[6] McDowell JM, Huang S, McKinney EC, An YQ, Meagher RB (1996). Structure and evolution of the actin gene family in Arabidopsis thaliana. Genetics.

[7] Meagher RB, McKinney EC, Vitale AV (1999). The evolution of new structures: Clues from plant cytoskeletal genes. Trends in Genetics.

[8] Slajcherová K, Fišerová J, Fischer L, Schwarzerová K (2012). Multiple actin isotypes in plants: diverse genes for diverse roles?. Front. Plant Sci..

[9] Chang M, Huang S (2015). Arabidopsis ACT11 modifies actin turnover to promote pollen germination and maintain the normal rate of tube growth. Plant J..

[10] Kandasamy MK, McKinney EC, Meagher RB (2009). A single vegetative actin isovariant overexpressed under the control of multiple regulatory sequences is sufficient for normal Arabidopsis development. Plant Cell.

[11] Kandasamy MK, McKinney EC, Meagher RB (2002). Functional nonequivalency of actin isovariants in Arabidopsis. Mol. Biol. Cell.

[12] Kandasamy MK, Burgos-Rivera B, McKinney EC, Ruzicka DR, Meagher RB (2007). Class-specific interaction of profilin and ADF isovariants with actin in the regulation of plant development. Plant Cell.

[13] Meagher RB, Kandasamy MK, McKinney EC (2008). Multicellular development and protein-protein interactions. Plant Signal. Behav..

[14] Kijima ST, Hirose K, Kong SG, Wada M, Uyeda TQP (2016). Distinct biochemical properties of Arabidopsis thaliana actin isoforms. Plant Cell Physiol..

[15] Kandasamy MK, McKinney EC, Meagher RB (1999). The late pollen-specific actins in angiosperms. Plant J..

[16] Kandasamy MK, Gilliland LU, McKinney EC, Meagher RB (2001). One plant actin isovariant, ACT7, is induced by auxin and required for normal callus formation. Plant Cell.

[17] Ren H (1997). Actin Purified from Maize Pollen Functions in Living Plant Cells. Plant Cell.

[18] Jing Y, Yi K, Ren H (2003). Actins from plant and animal sources tend not to form heteropolymers *in vitro* and function differently in plant cells. Protoplasma.

[19] Buchanan, B. B., Gruissem, W. & Jones, R. L. *Biochemistry and Molecular Biology of Plants*, *2nd Edition*. (Wiley, 2015).

[20] Westphal M (1997). Microfilament dynamics during cell movement and chemotaxis monitored using a GFP-actin fusion protein. Curr. Biol..

[21] Choidas A (1998). The suitability and application of a GFP-actin fusion protein for long-term imaging of the organization and dynamics of the cytoskeleton in mammalian cells. Eur. J. Cell Biol..

[22] Fischer M, Kaech S, Knutti D, Matus A (1998). Rapid actin-based plasticity in dendritic spines. Neuron.

[23] Liu AX, Zhang SBin, Xu XJ, Ren DT, Liu GQ (2004). Soluble expression and characterization of a GFP-fused pea actin isoform (PEAc1). Cell Res..

[24] Lo Y-S (2011). Actin in mung bean mitochondria and implications for its function. Plant Cell.

[25] Kost B, Spielhofer P, Chua NH (1998). A GFP-mouse talin fusion protein labels plant actin filaments *in vivo* and visualizes the actin cytoskeleton in growing pollen tubes. Plant J..

[26] Sheahan MB, Staiger CJ, Rose RJ, McCurdy DW (2004). A green fluorescent protein fusion to actin-binding domain 2 of Arabidopsis fimbrin highlights new features of a dynamic actin cytoskeleton in live plant cells. Plant Physiol..

[27] Era, a. *et al*. Application of Lifeact Reveals F-Actin Dynamics in Arabidopsis thaliana and the Liverwort, Marchantia polymorpha. *Plant Cell Physiol*. **50**, 1041–1048 (2009). 10.1093/pcp/pcp055 PMC2694730 19369273

[28] Du F, Ren H (2011). Development and application of probes for labeling the actin cytoskeleton in living plant cells. Protoplasma.

[29] Dyachok J, Sparks JA, Liao F, Wang YS, Blancaflor EB (2014). Fluorescent protein-based reporters of the actin cytoskeleton in living plant cells: Fluorophore variant, actin binding domain, and promoter considerations. Cytoskeleton.

[30] Vandekerckhove J, Weber K (1978). Mammalian cytoplasmic actins are the products of at least two genes and differ in primary structure in at least 25 identified positions from skeletal muscle actins. Proc. Natl. Acad. Sci. USA.

[31] Nagasaki A (2017). The Position of the GFP Tag on Actin Affects the Filament Formation in Mammalian Cells. Cell Struct. Funct..

[32] Gunning PW, Ghoshdastider U, Whitaker S, Popp D, Robinson RC (2015). The evolution of compositionally and functionally distinct actin filaments. J. Cell Sci..

[33] Dong XJ, Nagai R, Takagi S (1998). Microfilaments anchor chloroplasts along the outer periclinal wall in Vallisneria epidermal cells through cooperation of P-FR and photosynthesis. Plant Cell Physiol..

[34] Kandasamy MK, Meagher RB (1999). Actin-organelle interaction: Association with chloroplast in Arabidopsis leaf mesophyll cells. Cell Motil. Cytoskeleton.

[35] Sakurai N, Domoto K, Takagi S (2005). Blue-light-induced reorganization of the actin cytoskeleton and the avoidance response of chloroplasts in epidermal cells of Vallisneria gigantea. Planta.

[36] Kadota A (2009). Short actin-based mechanism for light-directed chloroplast movement in Arabidopsis. Proc. Natl. Acad. Sci. USA.

[37] Krzeszowiec W, Rajwa B, Dobrucki J, Gabryś H (2007). Actin cytoskeleton in Arabidopsis thaliana under blue and red light. Biol. cell.

[38] Oikawa, K. *et al*. Chloroplast unusual positioning1 is essential for proper chloroplast positioning. *Plant Cell* **15**, 2805–2815 (2003). 10.1105/tpc.016428 PMC282804 14615600

[39] Rocchetti A, Hawes C, Kriechbaumer V (2014). Fluorescent labelling of the actin cytoskeleton in plants using a cameloid antibody. Plant Methods.

[40] Wang P, Hussey PJ (2017). NETWORKED 3B: A novel protein in the actin cytoskeleton-endoplasmic reticulum interaction. J. Exp. Bot..

[41] Mitra R (2003). The Potato virus X TGBp2 protein association with the endoplasmic reticulum plays a role in but is not sufficient for viral cell-to-cell movement. Virology.

[42] Riedl J (2008). Lifeact: a versatile marker to visualize F-actin. Nat. Methods.

[43] Uyeda TQP, Iwadate Y, Umeki N, Nagasaki A, Yumura S (2011). Stretching actin filaments within cells enhances their affinity for the myosin II motor domain. PLoS One.

[44] Ngo KX (2016). Allosteric regulation by cooperative conformational changes of actin filaments drives mutually exclusive binding with cofilin and myosin. Sci. Rep..

[45] Doyle T, Botstein D (1996). Movement of yeast cortical actin cytoskeleton visualized *in vivo*. PNAS.

[46] Wu J-Q, Pollard TD (2005). Counting cytokinesis proteins globally and locally in fission yeast. Science.

[47] Staiger CJ (2016). MAPping the Function of Phytopathogen Effectors. Cell Host Microbe.

[48] Porter K, Day B (2016). From filaments to function: The role of the plant actin cytoskeleton in pathogen perception, signaling and immunity. J. Integr. Plant Biol..

[49] Henty-Ridilla JL (2013). The plant actin cytoskeleton responds to signals from microbe-associated molecular patterns. PLoS Pathog..

[50] Kang Y (2014). HopW1 from Pseudomonas syringae Disrupts the Actin Cytoskeleton to Promote Virulence in Arabidopsis. PLoS Pathog..

[51] Chaudhry F, Guérin C, von Witsch M, Blanchoin L, Staiger CJ (2007). Identification of Arabidopsis cyclase-associated protein 1 as the first nucleotide exchange factor for plant actin. Mol. Biol. Cell.

[52] Jásik J (2016). Actin3 promoter reveals undulating F-actin bundles at shanks and dynamic F-actin meshworks at tips of tip-growing pollen tubes. *Plant Signal*. Behav..

[53] Qu X (2013). *Arabidopsis* Villins Promote Actin Turnover at Pollen Tube Tips and Facilitate the Construction of Actin Collars. Plant Cell.

[54] Tseng PC, Pollard TD (1982). Mechanism of action of Acanthamoeba profilin: demonstration of actin species specificity and regulation by micromolar concentrations of MgCl2. J. Cell Biol..

[55] Cruz DL, Cofilin EM (2005). binding to muscle and non-muscle actin filaments: isoform-dependent cooperative interactions. J. Mol. Biol..

[56] Müller M (2013). Distinct functional interactions between actin isoforms and nonsarcomeric myosins. PLoS One.

[57] Hayden SM, Miller PS, Brauweiler A, Bamburg JR (1993). Analysis of the interactions of actin depolymerizing factor with G- and F-actin. Biochemistry.

[58] Pantaloni D, Carlier MF (1993). How profilin promotes actin filament assembly in the presence of thymosin beta 4. Cell.

[59] Voigt B (2005). GFP-FABD2 fusion construct allows *in vivo* visualization of the dynamic actin cytoskeleton in all cells of Arabidopsis seedlings. Eur. J. Cell Biol..

[60] Washington RW, Knecht DA (2008). Actin binding domains direct actin-binding proteins to different cytoskeletal locations. BMC Cell Biol..

[61] Belin BJ, Goins LM, Mullins RD (2014). Comparative analysis of tools for live cell imaging of actin network architecture. Bioarchitecture.

[62] Ketelaar T, Anthony RG, Hussey PJ (2004). Green fluorescent protein-mTalin causes defects in actin organization and cell expansion in Arabidopsis and inhibits actin depolymerizing factor’s actin depolymerizing activity *in vitro*. Plant Physiol..

[63] Cvrčková F, Oulehlová D (2017). A new kymogram-based method reveals unexpected effects of marker protein expression and spatial anisotropy of cytoskeletal dynamics in plant cell cortex. Plant Methods.

[64] van der Honing HS, van Bezouwen LS, Emons AMC, Ketelaar T (2011). High expression of Lifeact in Arabidopsis thaliana reduces dynamic reorganization of actin filaments but does not affect plant development. Cytoskeleton.

[65] Kong SG (2006). Blue light-induced association of phototropin 2 with the Golgi apparatus. Plant J..

[66] Jouanneau J, Péaud-Lenoël C (1967). Growth and synthesis of proteins in cell suspensions of kinetin dependent tobacco. Physiol. Plant..

[67] Axelos M, Curie C, Mazzolini L, Bardet C, Lescure B (1992). A protocol for transient gene expression in Arabidopsis thaliana protoplasts isolated from cell suspension cultures. Plant Physiol. Biochem..

[68] Yamada H (2004). Rapid response of Arabidopsis T87 cultured cells to cytokinin through His-to-Asp phosphorelay signal transduction. Biosci. Biotechnol. Biochem..

[69] Yoo S-D, Cho Y-H, Sheen J (2007). Arabidopsis mesophyll protoplasts: a versatile cell system for transient gene expression analysis. Nat. Protoc..

[70] Goodin MM, Dietzgen RG, Schichnes D, Ruzin S, Jackson AO (2002). pGD vectors: versatile tools for the expression of green and red fluorescent protein fusions in agroinfiltrated plant leaves. Plant J..

[71] Spudich JA, Watt S (1971). The regulation of rabbit skeletal muscle contraction. I. Biochemical studies of the interaction of the tropomyosin-troponin complex with actin and the proteolytic fragments of myosin. J. Biol. Chem..

