# Supplementary material for: Arabidopsis vegetative actin isoforms, AtACT2 and AtACT7, generate distinct filament arrays in living plant cells
Source: Sci Rep. 2018 Mar 12;8:4381. doi: 10.1038/s41598-018-22707-w (PMC5847576; doi:10.1038/s41598-018-22707-w)
Supplement: Supplementary file 1 — Supplementary Information [file 41598_2018_22707_MOESM1_ESM.pdf]

Supplementary information for:

Arabidopsis vegetative actin isoforms, AtACT2 and AtACT7, generate distinct filament arrays in living plant cells

Saku T. Kijima, Christopher J. Staiger, Kaoru Katoh, Akira Nagasaki, Kohji Ito and Taro Q.P. Uyeda

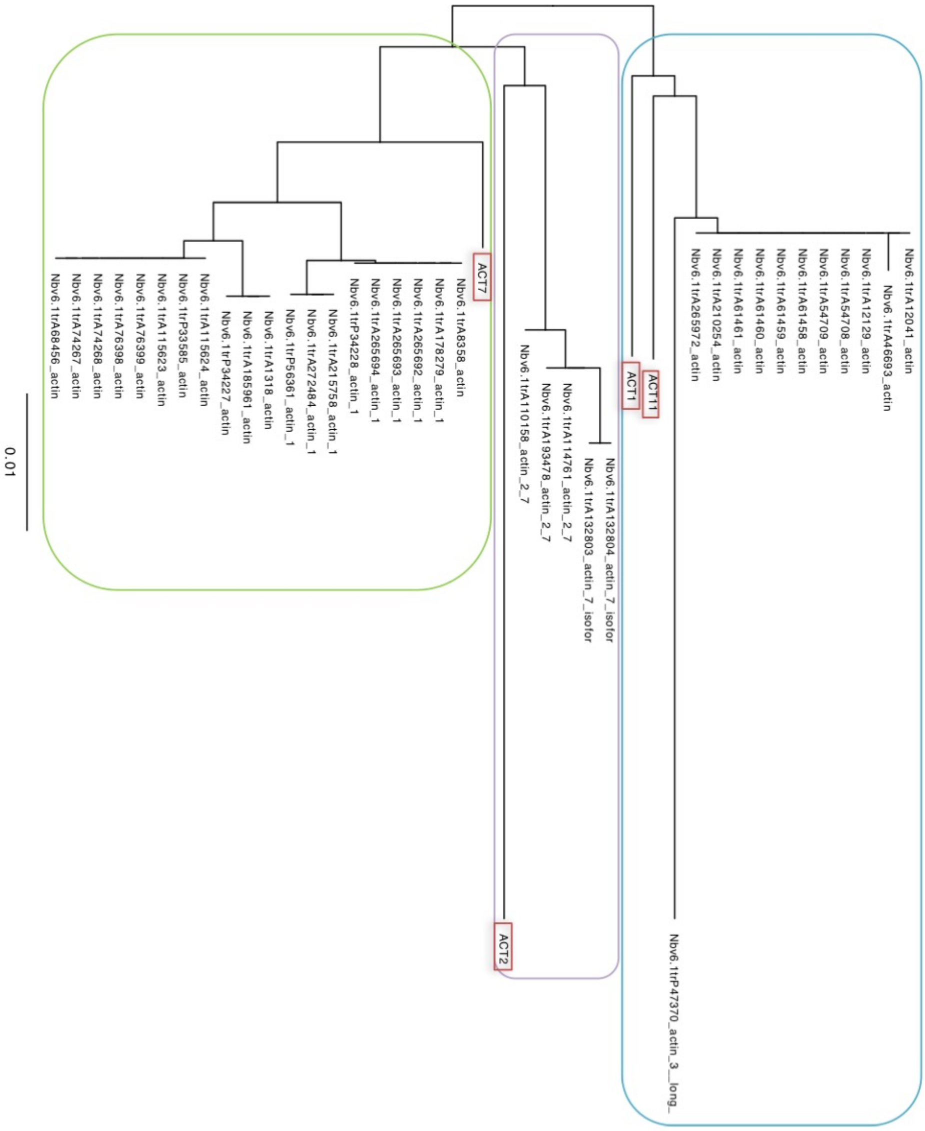

Supplemental Figure 1. Phylogenetic relationships among *A. thaliana* and *N. benthamiana* actin isoforms. Red boxes indicate major *A. thaliana* actin isoforms. All others are *N. benthamiana* actin isoforms. These actin isoforms are grouped into three major clades, one containing *Arabidopsis* reproductive actin (blue square), one containing vegetative AtACT2 (purple square) and one containing vegetative AtACT7 (green square). This phylogenetic tree was constructed using Clustal W (DDBJ) website (http://clustalw.ddbj.nig.ac.jp) and visualized by Fig Tree 1.4.3. The accession numbers and amino acid sequences of *N. benthamiana* actin were acquired from QUT *Nicotiana benthamiana* Genome & Transcriptome (http://benthgenome.qut.edu.au). Individual accession numbers of *A. thaliana* actins, ACT1, ACT2, ACT7 and ACT11 are AT2G37620, AT3G18780, AT5G09810 and AT3G12110, respectively.

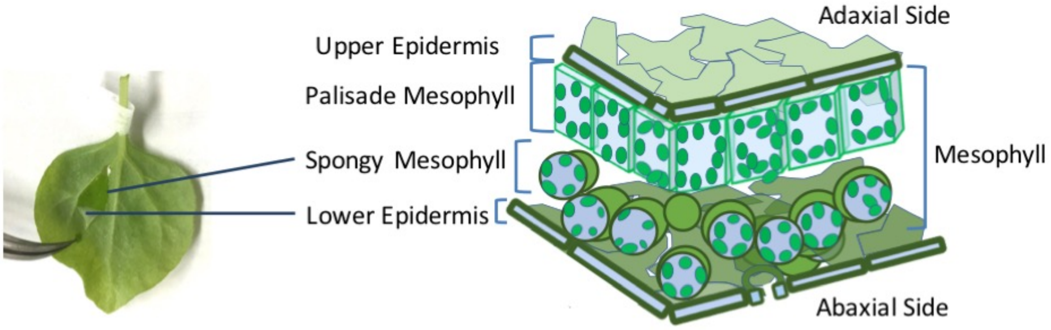

Supplemental Figure 2. Schematic illustrations of the leaf structure. We observed leaf spongy mesophyll cells by peeling off a thin layer of epidermis. When observing leaf epidermal cells, the epidermis on the abaxial side of the leaf was observed.

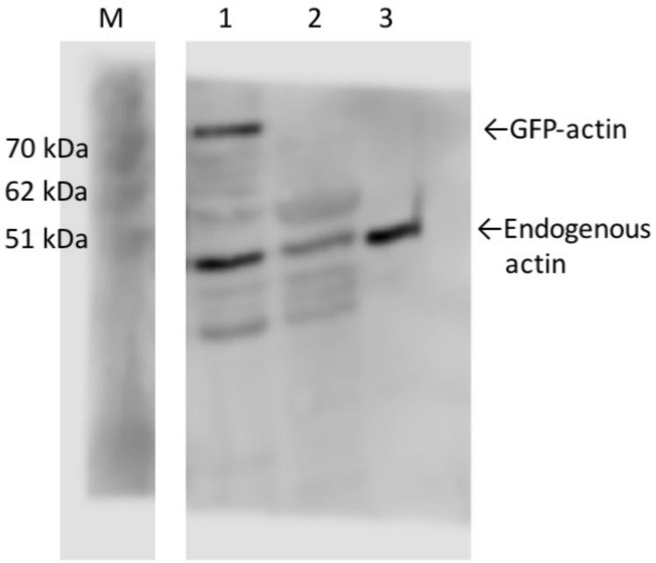

Supplemental Figure 3. Expression levels of GFP-actin and endogenous actin in a leaf 40 hr after infiltration. Western blot analysis of the soluble fraction in extraction buffer (25 mM Tris pH 7.5, 10 mM NaCl, 10 mM $MgCl_2$, 5 mM EDTA, protease inhibitors) after homogenizing leaf cells. Primary antibody was anti-actin antibody (MAB1501R; Chemicon, CA, USA). Lane M is the marker. Lane 1 is the soluble fraction of a leaf expressing GFP-AtACT2. Lane 2 is the soluble fraction of a non-transfected leaf. Lane 3 is purified skeletal muscle actin.

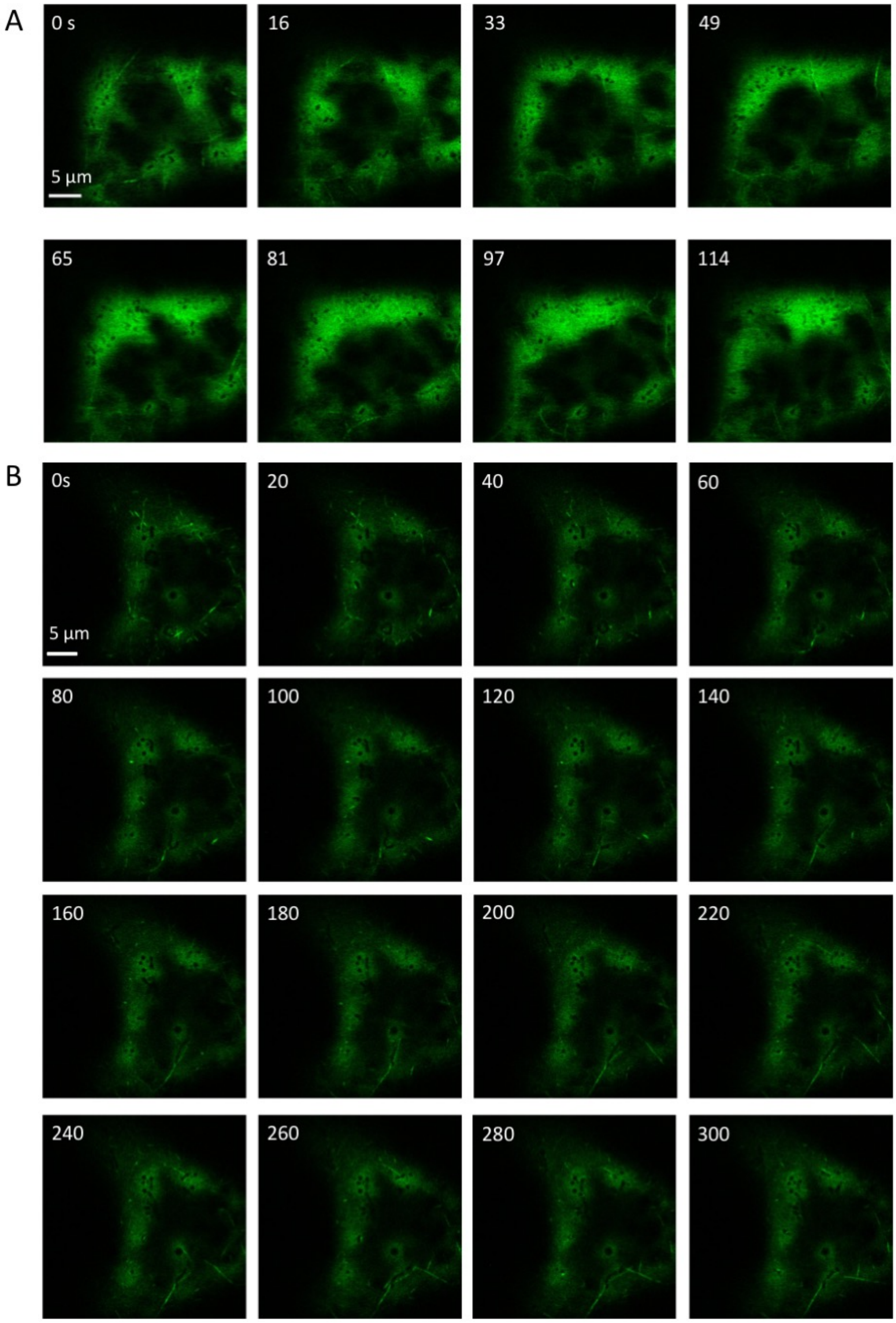

Supplemental Figure 4. Time-lapse imaging of AtACT2 and AtACT7 in *N. benthamiana* leaf epidermal cells. (A) Time-lapse optical section images of GFP-AtACT2 at 16 s intervals. (B) Time-lapse optical section images of GFP-AtACT7 at 20 s intervals.

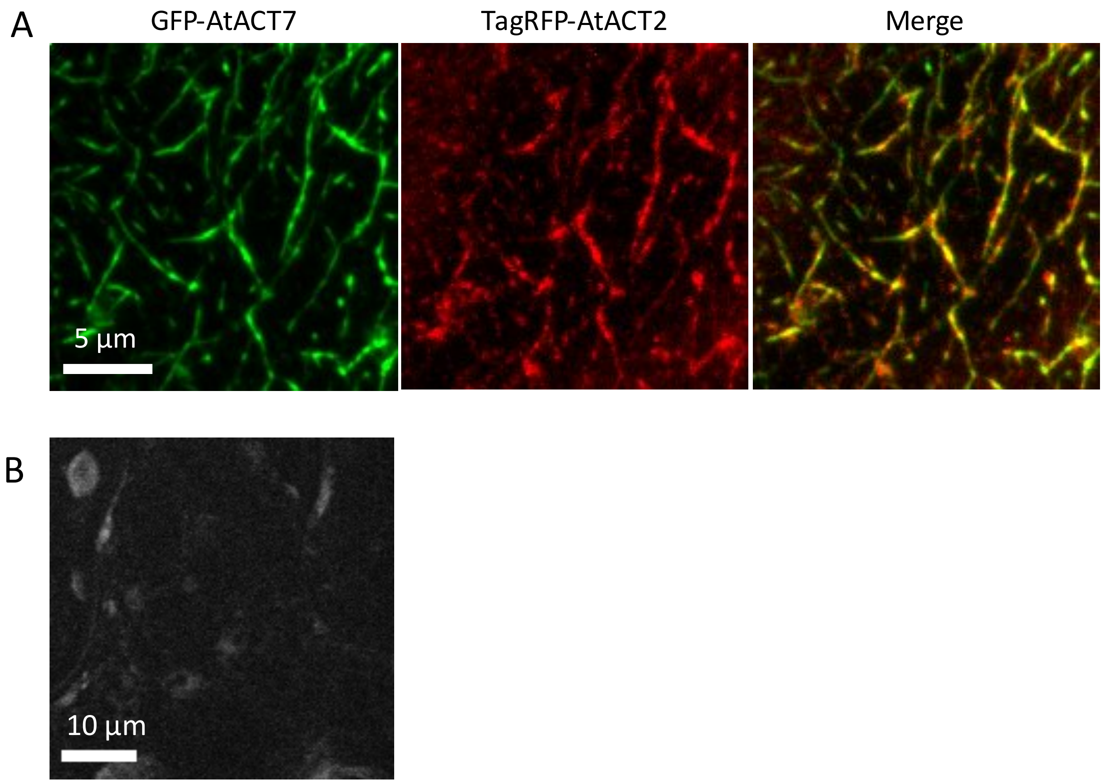

Supplemental Figure 5. Distribution of AtACT2 and AtACT7 in fixed *N. benthamiana* leaf epidermal cells. (A) Z-series projections of a leaf epidermal cell transiently co-expressing GFP-AtACT2 and TagRFP-AtACT7, and fixed by infiltration of fixation buffer 1 [200 µM MBS (*m*-maleimidobenzoyl-*N*-hydroxysuccinimide ester), 0.2% PLURONIC F-127, 50 mM PIPES pH 6.8, 10 mM EGTA, 5 mM $MgCl_2$, 50 mM KCl] into the leaf tissue. After incubation for 15 min, the surface of the leaf was rinsed with actin stabilization buffer [50 mM PIPES pH 6.8, 10 mM EGTA, 5 mM $MgCl_2$, 50 mM KCl] and observed in this buffer. (B) Z-series projections of a leaf epidermal cell transiently expressing GFP-AtACT7, and fixed by formaldehyde. The leaf tissue was fixed by infiltration of fixation buffer 2 [2% paraformaldehyde, 1% dimethyl sulfoxide (DMSO), 50 mM PIPES pH 6.4, 10 mM EGTA, 5 mM $MgCl_2$] and incubated for 60 min, followed by infiltration of Triton buffer (0.1% Triton X-100, 50 mM PIPES pH 6.4, 10 mM EGTA, 5 mM $MgCl_2$). The surface of the leaf was rinsed with fixation buffer 2 without paraformaldehyde and DMSO. No filamentous structures were observed under this condition. This is consistent with the previous result that actin filaments in *N. tabacum* BY2 cells were not well preserved by aldehyde fixatives [1], although aldehyde fixation was successfully employed to immunostain actin structures in root cells [2].

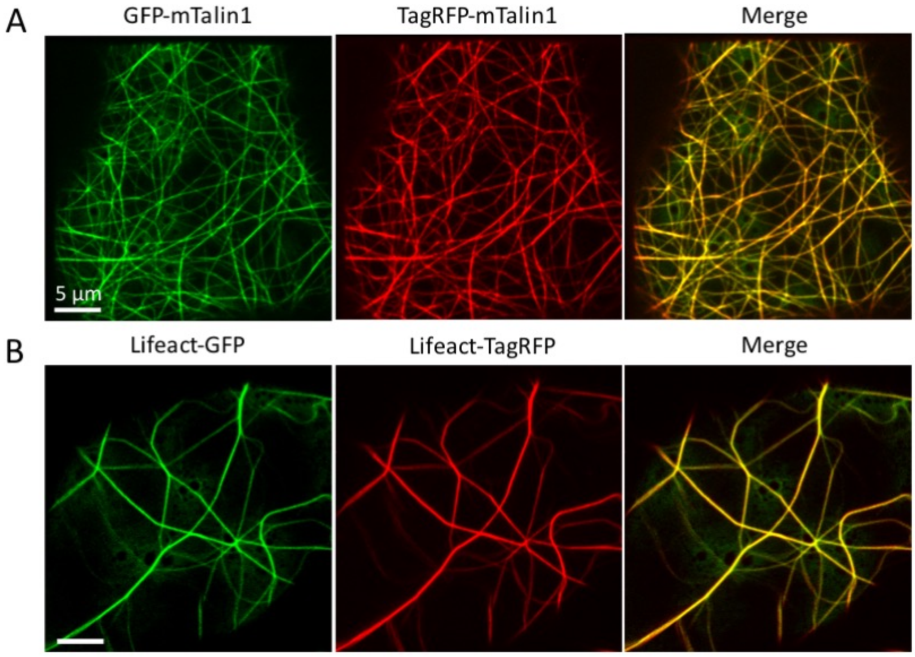

Supplemental Figure 6. Distribution of two generic actin filament probes fused with different fluorescent proteins in *N. benthamiana* leaf epidermal cells. (A) Z-series projections of leaf epidermal cells transiently co-expressing GFP-mTalin1 (top left) and TagRFP-mTalin1 (top middle). The merged image is shown on the top right. (B) Z-series projections of leaf epidermal cells transiently co-expressing Lifeact-GFP (bottom left) and Lifeact-TagRFP (bottom middle). The merged image is shown on the bottom right.

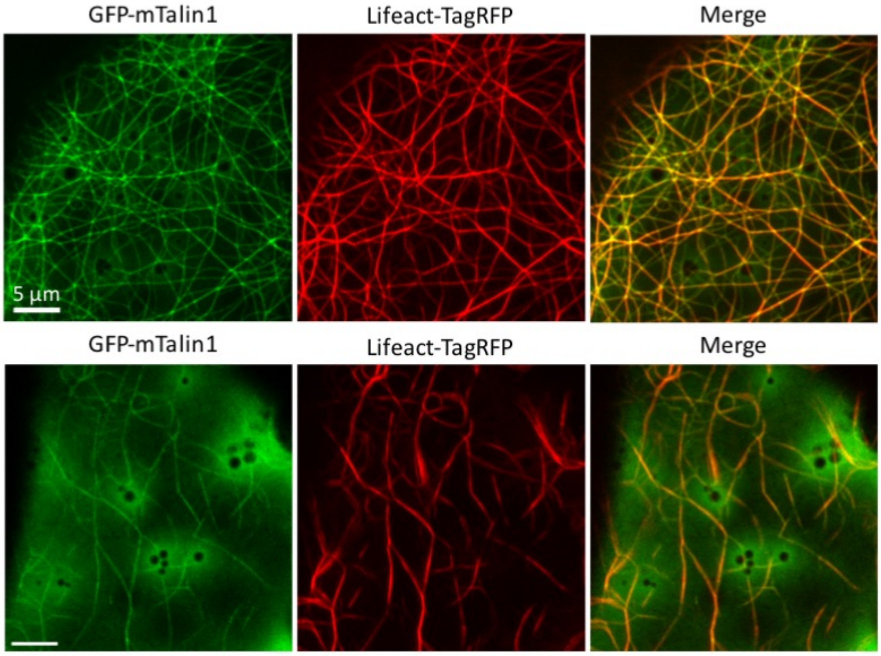

Supplemental Figure 7. Distribution of GFP-mTalin1 and Lifeact-TagRFP in *N. benthamiana* leaf epidermal cells. Z-series projections of leaf epidermal cells transiently co-expressing GFP-mTalin1 (left) and Lifeact-TagRFP (middle). The merged image is shown on the right. Some cells showed thin and dense filament arrays, namely, the mTalin1 type (upper row), while others showed thick and sparse filament arrays, namely, the Lifeact type (lower row).

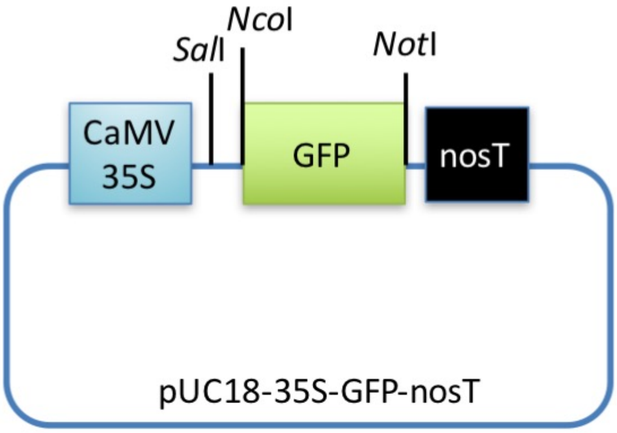

Supplemental Figure 8. Expression vector pUC18-35S-GFP-nosT. This expression vector was a kind gift from Sam-Geun Kong. *Sal*I, *Nco*I and *Not*I restriction enzyme sites were used in the cloning experiments in this study.

Reference

1. Sonobe, S. & Shibaoka, H. Cortical fine actin filaments in higher plant cells visualized by rhodamine-phalloidin after pretreatment with m-maleimidobenzoyl N-hydroxysuccinimide ester. *Protoplasma* **148,** 80–86 (1989).
2. Vitha, S., Baluška, F., Braun, M., Šamaj, J., Volkmann, D. & Barlow, P. W. Comparison of cryofixation and aldehyde fixation for plant actin immunocytochemistry: aldehydes do not destroy F-actin. *Histochem. J.* **32,** 457–466 (2000).
